# Echinocandins have an alternative mode of action on biomimetic membranes that is not directly related to the functioning of (1,3) beta-glucan synthase

**DOI:** 10.1038/s41420-026-03133-8

**Published:** 2026-04-28

**Authors:** Anna I. Malykhina, Svetlana S. Efimova, Natalia E. Grammatikova, Anna N. Tevyashova, Andrey E. Shchekotikhin, Olga S. Ostroumova

**Affiliations:** 1 Institute of Cytology of Russian Academy of Sciences, Saint Petersburg, Russian Federation; 2 Gause Institute of New Antibiotics, Moscow, Russian Federation; 3 School of Science, Constructor University, Bremen, Germany

**Keywords:** Fungal infection, Antimicrobial resistance

## Abstract

Echinocandins are the preferred agents for treating invasive candidiasis; however, rising resistance in *Candida* species poses a significant challenge for patient care. This study aimed to improve the efficacy of echinocandins against clinical *Candida* isolates by enhancing their noncanonical membrane activity. The mechanisms of action of anidulafungin, caspofungin, and micafungin on lipid membranes were investigated using a range of biophysical methods and molecular dynamics approaches. Antifungal activity was assessed using a panel of clinical *Candida* isolates. The results indicated that echinocandins exhibit greater selectivity for ergosterol-containing membranes than for cholesterol-enriched membranes. Echinocandins caused differential phase disordering, leading to a significant increase in the size of sterol-rich ordered domains, induced membrane stress/perturbation, and promoted the formation of ion-permeable transmembrane pores. The membrane activity of echinocandins was enhanced upon incorporation into liposomes. Echinocandin liposomes exhibited reduced MICs against clinical *Candida* isolates (down to 0.002 µg/mL) compared with conventional echinocandins. This reduction in MIC was observed regardless of the strain’s susceptibility to standard echinocandins. Enhancing the membrane activity of echinocandins through their incorporation into liposomal formulations represents a promising strategy to improve antifungal efficacy and address the increasing resistance observed in clinical *Candida* isolates.

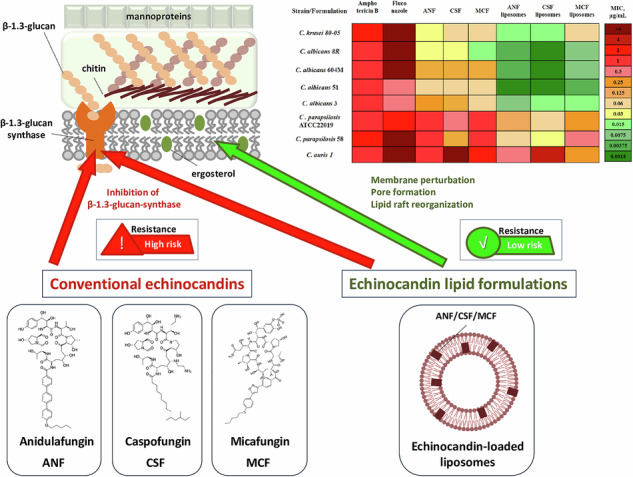

## Introduction

Fungi that cause disease pose a significant risk to human well-being because of their increasing prevalence and growing resistance to medications [1]. Invasive fungal infections often affect critically ill patients and those with immunodeficiency, leading to high morbidity and mortality rates [2, 3]. The formation of biofilms by many pathogenic fungi also significantly complicates antifungal treatment, particularly in immunocompromised patients [4]. For example, *Candida* spp. are now ranked third among microorganisms associated with catheter-related bloodstream infections [5]. In 2022, the World Health Organization published a list of critical and high-priority fungal pathogens, including *Candida auris*, *C. albicans*, *C. glabrata*, and *C. parapsilosis*, and called on academia and industry “to strengthen the global response to fungal infections and antifungal resistance” [6].

Echinocandins are semisynthetic cyclic lipopeptides that represent a new class of antifungal drugs, recommended as first-line therapy by the Infectious Diseases Society of America [7]. They are highly effective in the treatment of invasive fungal infections [8] and against fungal biofilms [9]. However, clinical failures are associated with emerging resistance, particularly among *Candida* spp. [10, 11]. Echinocandins noncompetitively inhibit (1,3)-β-glucan synthase (GS), leading to disturbances in fungal cell wall synthesis [12]. The structure of this transmembrane protein was recently determined using cryo-electron microscopy in two independent studies [13, 14]. Both studies established that echinocandin-resistant mutations cluster in hotspot regions of the transmembrane helices and are closely related to the membrane environment. Hu et al. [13] noted that one GS mutation (S643P) can alter the bound lipid molecules surrounding hotspot region 1 and reasoned that the echinocandin binding site lies within the membrane segment of GS or that the drugs act through membrane changes. Zhao et al. [14] proposed that echinocandins likely inhibit glucan translocation by acting on transporting channels in the membrane. Moreover, this group found that bound lipids copurified with GS contained endogenous ergosterol (Erg), which assisted in the structural stabilization of GS. The three-dimensional structure revealed by docking studies showed that GS-bound lipids anchored the echinocandin tail in a binding pocket through hydrophobic interactions [15].

Undoubtedly, the fungicidal effect of echinocandins is related to GS inhibition. However, some non-GS-related mutations are known to lead to the development of resistance to echinocandins. Mutations in genes encoding enzymes involved in sphingolipid biosynthesis result in caspofungin (CSF) resistance in *C. glabrata* [16, 17]. Mutations in four different genes produced the same changes in membrane composition, namely the accumulation of the long-chain bases dihydrosphingosine and phytosphingosine; the external addition of these substances triggered similar resistance. Furthermore, Satish et al. [18] demonstrated that stress-induced echinocandin resistance in *Aspergillus fumigatus* produced similar membrane changes and that exogenous dihydrosphingosine and phytosphingosine added to purified GS inhibited its catalytic activity.

A recent screening study of CSF-sensitive strains of *Cryptococcus neoformans* identified notable mutations responsible for this sensitivity [19]. Two of the four identified deletions occurred in genes involved in Erg synthesis and transport, leading to elevated susceptibility to CSF at higher temperatures without obvious cell wall defects. Moreover, microscopy showed that the underlying cause of this sensitivity is a loss of membrane integrity, which may either increase permeability to CSF or change the location or orientation of GS, thereby making it more susceptible to inhibition [19]. In another study, cryo-electron tomography and live-cell imaging revealed that GS is nonhomogeneously distributed in the plasma membrane and localized in distinct Erg-enriched microdomains, presumably the membrane compartment occupied by the arginine permease Can1 (MCC). These microdomains are disrupted by CSF application, resulting in GS inhibition [20, 21]. Together with the structural studies mentioned above, these findings indicate that membrane properties are extremely important for proper GS function. However, the direct influence of echinocandins on target cell membranes has not yet been studied.

Although echinocandins are generally well tolerated, they have two major drawbacks that limit their use. The first is the presence of natural resistance to these antibiotics in some fungi. For example, Basidiomycetes, Mucoromycetes, and *Fusarium* species exhibit resistance to echinocandins because β-1,3-glucan constitutes only a minor component of their cell walls [22]. Second, fungal variants with acquired resistance can emerge rapidly. For example, it took only a decade for *C. auris*, a multidrug-resistant yeast that causes invasive infections with high mortality rates, to spread worldwide [23]. It has been reported that 93% of isolates of this pathogen were resistant to fluconazole, 35% to amphotericin B, and 7% to echinocandins [24]. Identification and enhancement of the direct action of echinocandins on fungal membranes may help circumvent pathogen resistance.

**Fig. 1:**
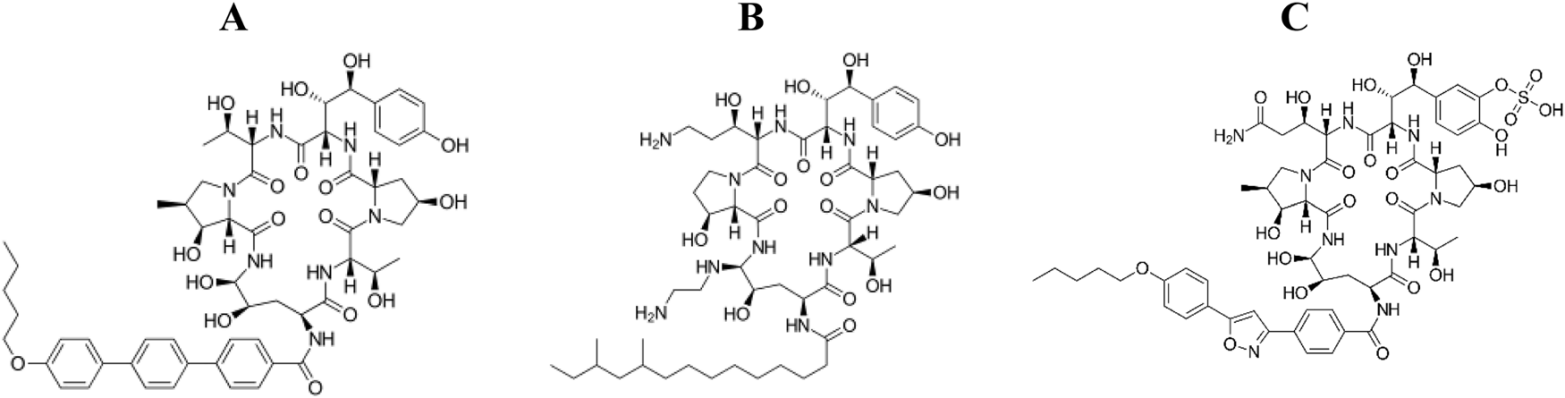
Echinocandins. Chemical structures of the tested echinocandins: **A** ANF, **B** CSF, and **C** MCF.

In this study, we implemented a comprehensive approach to investigate the mode of action of the echinocandins anidulafungin (ANF), CSF, and micafungin (MCF) (Fig. 1) on model lipid membranes. The approach included differential scanning microcalorimetry (DSmC) of lipid phase transitions, confocal fluorescence microscopy of lipid-ordered domains, fluorimetry of calcein leakage from lipid vesicles, electrophysiological registration of ionic currents through planar lipid bilayers, and molecular dynamics (MD) simulations of echinocandin-treated lipid bilayers. The main findings highlight the disordering effect, alterations in the phase segregation scenario, and pore formation induced by echinocandins. These effects depended on the type of echinocandin and the sterol composition of the target membranes. The antimicrobial activity of echinocandin-loaded liposomes was assessed against a wide range of *Candida* spp., including fluconazole- and echinocandin-resistant strains. Our results reveal a possible alternative mechanism for the antifungal action of echinocandins that may involve alteration of the lipid microenvironment of GS and increased target membrane permeability. An equally important finding was that the antifungal activity of echinocandins increased significantly when delivered in liposomal form.

## Results

### Echinocandins affect membrane lipid phase organization

**Fig. 2:**
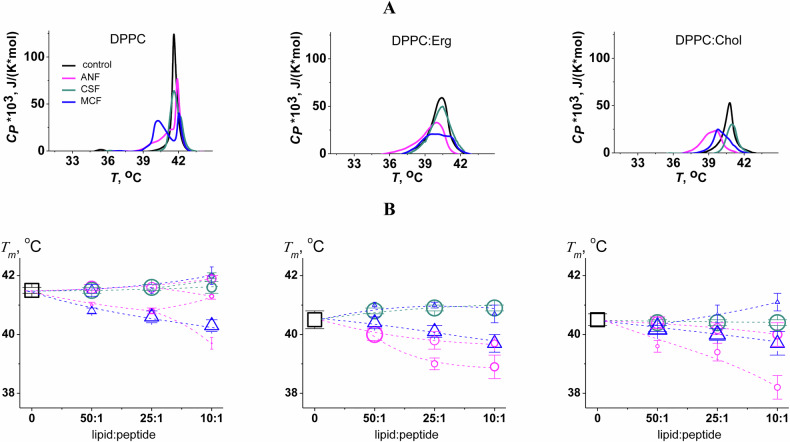
Effects of echinocandins on lipid melting. **A** Heating thermograms of DPPC (*left panel*), DPPC:Erg (85:15 mol.%) (*middle panel*), and DPPC:Chol (85:15 mol.%) (*right panel*) liposomes in the absence (control, *black curves*) and presence of ANF (*magenta curves*), CSF (*dark cyan curves*), and MCF (*blue curves*) at lipid:echinocandin ratios of 10:1. **B** Dependence of melting temperatures of different subphases on the lipid:echinocandin molar ratio. The size of the symbols is proportional to the component’s contribution to the enthalpy change.

DSmC of unilamellar lipid vesicles was used to investigate echinocandin-induced alterations in the phase behavior of membrane lipids. Lipid vesicles were prepared from pure DPPC or mixtures of DPPC and Erg or Chol to mimic fungal and mammalian membranes, respectively [25–28]. In the absence of echinocandins, the melting temperature (*T*_*m*_) of DPPC, DPPC:Erg (85:15 mol.%), and DPPC:Chol (85:15 mol.%) was 41.5 °C ± 0.1 °C, 40.5 °C ± 0.1 °C, and 40.6 °C ± 0.2 °C, respectively. The widths of the peaks related to the main transition (∆*T*_*b*_) of DPPC, DPPC:Erg, and DPPC:Chol were 2.6 °C ± 0.3 °C, 4.9 °C ± 0.2 °C, and 3.8 °C ± 0.2 °C, respectively. The results obtained for DPPC and its mixtures with sterols were in good agreement with the literature data [29, 30]. The tested echinocandins affected the thermotropic behavior of membrane lipids. Figure 2A shows representative heating thermograms of DPPC, DPPC:Erg, and DPPC:Chol vesicles before and after the addition of various echinocandins to the liposome suspension at a lipid:drug molar ratio of 10:1. At lipid:drug molar ratios of 50:1 and lower, ANF, CSF, and MCF completely abolished the gel-to-ripple phase transition of pure DPPC, which was observed at 35.1 °C ± 0.5 °C in the absence of echinocandins (Fig. 2A). The molecular origin of ripple-phase formation is associated with changes in the DPPC headgroup region, and adsorption of echinocandins upsets the balance between polar and hydrocarbon volumes. ANF and MCF broadened the melting peaks of DPPC, DPPC:Erg, and DPPC:Chol, whereas CSF elevated ∆*T*_*b*_ only in the DPPC model (Fig. 2A). The increase in the width of the transition peak might indicate a loss of cooperativity of the phase transition and a subsequent decrease in the cooperative lipid unit. It should also be noted that echinocandins, especially CSF, diminished the enthalpy of melting. Figure S1 (Supplementary Information) presents the dependence of the relative alteration in enthalpy (*δH*) on the lipid:drug molar ratio. The enthalpy losses at lower lipid:echinocandin ratios might be related to the ability of the lipopeptides to form mixed lipid–echinocandin micelles. More likely, the micelle-forming efficiency of CSF is higher than that of the other two echinocandins because of differences in the structure of their tails and the charge of their head groups (Fig. 1).

Moreover, the main peaks in the thermograms of all lipid systems in the presence of ANF and MCF acquired complex profiles characterized by two or three overlapping components (Fig. 2A). CSF had a similar effect on DPPC. This observation may indicate the existence of several lipid phases. To assess the contribution of the various subphases, decomposition–deconvolution analysis of the DPPC, DPPC:Erg, and DPPC:Chol main transition peaks in the presence of various echinocandins was performed, and the temperature of the maximum and the contribution of each component to the main transition enthalpy change were determined. Figure 2B presents the dependence of the maximum temperatures of different subphases (Δ*T*_*m_i*_) on the lipid:drug molar ratio. The size of the symbols is proportional to the component’s contribution to the enthalpy change. As a rule, the contribution of the subphase with the higher melting temperature (subphase #1) decreased, whereas that of the component with the lower transition point (subphase #2) increased as the lipid:drug molar ratio decreased. At lipid:ANF molar ratios of 10:1, an additional intermediate phase (subphase #3) appeared. In the case of DPPC, subphase #1 was characterized by the same melting temperature (at lipid:drug molar ratios of 50:1 and 25:1) or even a higher melting temperature (at a lipid:drug molar ratio of 10:1) compared with the control value (Fig. 2B); for this reason, it may be attributed to the melting of a practically pure lipid phase. The increase in the melting temperature of DPPC at low lipid:echinocandin molar ratios might be interpreted as an effect of bilayer dehydration [31] due to antibiotic adsorption onto the membrane surface. In support of this interpretation, MD data indicated a decrease in the number of hydrogen bonds between the DPPC membrane and water in the presence of ANF, CSF, and MCF by approximately 2%, 1%, and 4% at 25 °C and 7%, 5%, and 8% at 50 °C, respectively. The other peak component(s) were related to lipid phases enriched with echinocandins. The decrease in the melting temperature and the sharpness of the lipid phase transition produced by ANF and MCF indicated their membrane-disordering effect. Thus, the incorporation and immersion of echinocandins into the bilayer were accompanied by an increase in lipid motional freedom and acyl-chain mobility, probably due to an increase in APL.

To demonstrate the growth in APL, we performed MD simulations. In total, 16 echinocandin molecules were initially embedded in a solvated lipid bilayer system with a lipid:drug molar ratio of 10:1. The simulations were run for 100 ns at 25 °C and 50 °C, which correspond to DPPC-containing membranes in the gel and fluid states, respectively. The mean APL values are presented in Table S1 (Supplementary Information). All tested echinocandins increased APL. Unexpectedly, the greatest effect was observed for CSF, regardless of the lipid phase state (gel vs fluid), the saturation of the phospholipid chains (DPPC vs POPC), or the type of sterol (Erg vs Chol) (Table S1, Supplementary Information), although this compound had no significant impact on the thermograms (Fig. 2A). This discrepancy can be explained by differences in the design of the MD and DSmC experiments, in which echinocandins were either embedded directly into membrane environments or incorporated into lipid bilayers from aqueous solution, respectively. To assess drug incorporation into the bilayers, we performed a series of MD simulations in which four echinocandin molecules were allowed to insert from solution into membranes composed of POPC and different sterols (Table S2, Supplementary Information). CSF showed a lower probability of insertion into Erg-enriched bilayers than did ANF and MCF. Moreover, CSF and MCF were not able to incorporate into Chol-containing membranes during the simulations, whereas ANF showed preferential selectivity toward Erg-enriched bilayers. Thus, the lower ability of CSF to integrate into membranes from aqueous solution compared with ANF and MCF, as well as the preferential selectivity of all antibiotics for Erg-containing membranes over Chol-enriched ones, was demonstrated. Additional DSmC experiments in which echinocandins were added during liposome preparation demonstrated a clear disordering effect of CSF and greater efficiency of ANF and MCF in decreasing the lipid transition temperature relative to that observed when echinocandins were incubated with aqueous dispersions of preformed liposomes (Fig. S2, Supplementary Information). These results clearly indicate the high potential of liposomal formulations of echinocandins compared with aqueous dilutions. Moreover, inclusion of Chol in liposomes may provide the required selectivity of the drug formulation toward Erg-containing fungal membranes.

Interestingly, in the DPPC and DPPC:Chol systems, the echinocandin-induced increase in APL at 25 °C (corresponding to membranes in the gel phase) was more pronounced than at 50 °C (liquid-crystalline state), whereas for DPPC:Erg it was virtually identical in the two phase states (Table S1, Supplementary Information). The increase in APL is mainly related to the expansion of the volume of the hydrophilic bilayer region. To assess echinocandin-induced perturbations in the hydrocarbon core, we evaluated changes in deuterium order parameters (S_CD_) using MD simulations (Fig. S3, Supplementary Information). As shown in Fig. S3, the ability of echinocandins to reduce lipid order parameters in DPPC membranes was more pronounced at 25 °C, when DPPC was in the gel phase, than at 50 °C, when the lipid was in the liquid-crystalline phase. The addition of sterols to the membranes altered the influence of echinocandins on S_CD_: the disordering effect became more pronounced at 50 °C than at 25 °C (Fig. S3, Supplementary Information).

**Fig. 3:**
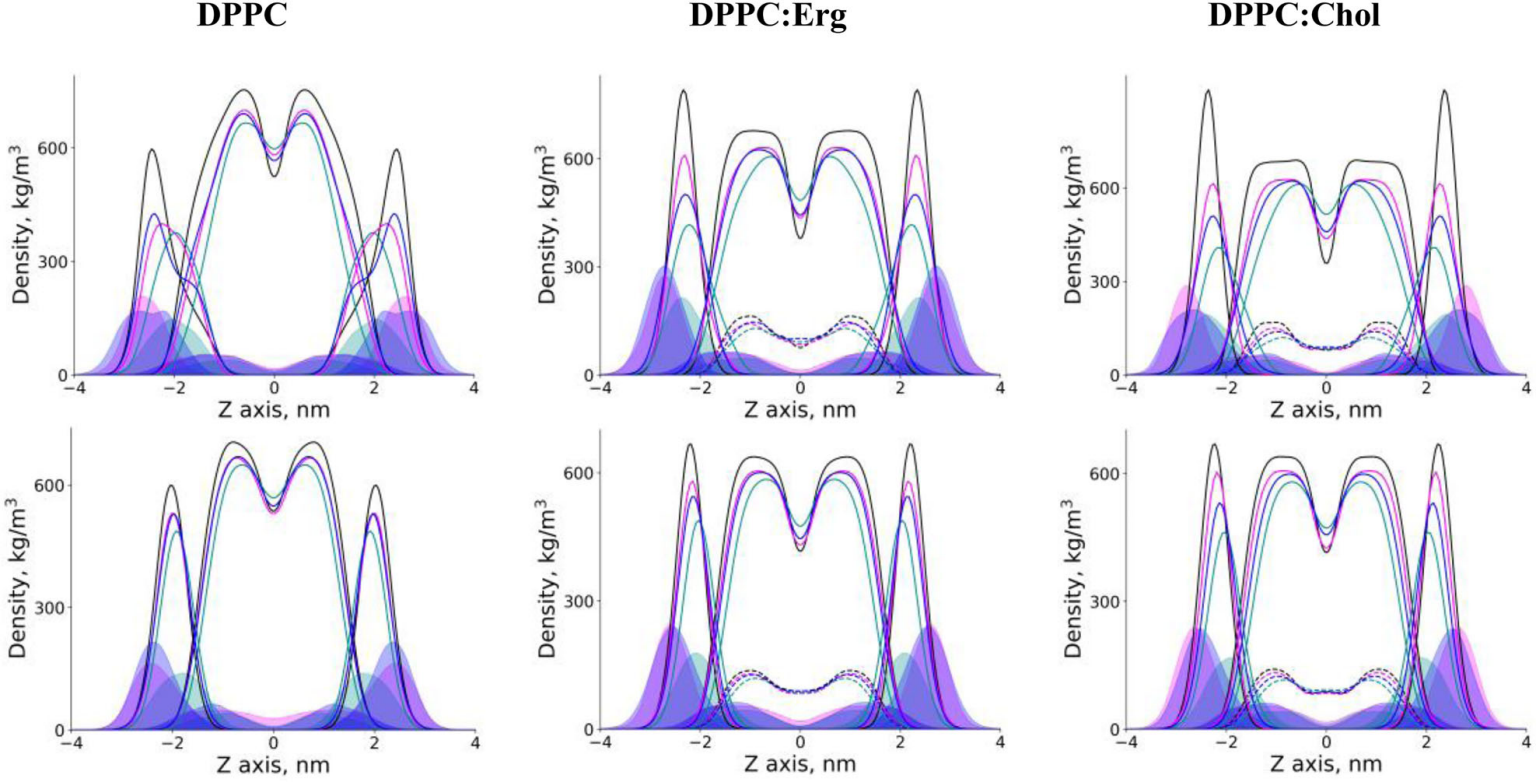
Density redistribution in the presence of echinocandins. Density plots for DPPC headgroups and acyl tails (solid lines), sterols (dashed lines), and echinocandins (colored areas) in membranes composed of DPPC (left panel), DPPC:Erg (85:15 mol.%) (middle panel), and DPPC:Chol (85:15 mol.%) (right panel) in the absence (control, black curves) and presence of ANF (magenta curves), CSF (dark cyan curves), and MCF (blue curves), at lipid:echinocandin ratios of 10:1, based on MD data at 25 °C (upper panel) and 50 °C (lower panel).

The density distributions of DPPC head groups and tails, sterols, and echinocandin heads and tails along the membrane normal (*z*-axis) for each system at two temperatures are shown in Fig. 3. An increase in density at *z* = 0 indicates that the tails of DPPC molecules from opposite leaflets become increasingly interdigitated in the presence of antibiotics. Echinocandins decreased the density maxima corresponding to DPPC heads and tails, although the density-reducing effect of the drugs was stronger in the hydrophilic region than in the hydrophobic core. The decrease in density likely results from the unbalanced volume-expanding effect of the antibiotics: the large polar peptide head produces a stronger effect than the single hydrophobic tail. Because the balance between head groups and tails determines spontaneous curvature [32–35], these results suggest that echinocandins induce positive curvature stress. Their curvature-modifying ability depended on the antibiotic type, sterol content, and simulation temperature, with the effect being weaker at 50 °C than at 25 °C. This indicates that in sterol-enriched bilayers containing antibiotics, spontaneous curvature varies less across different lipid phase states than in membranes lacking these agents.

**Fig. 4:**
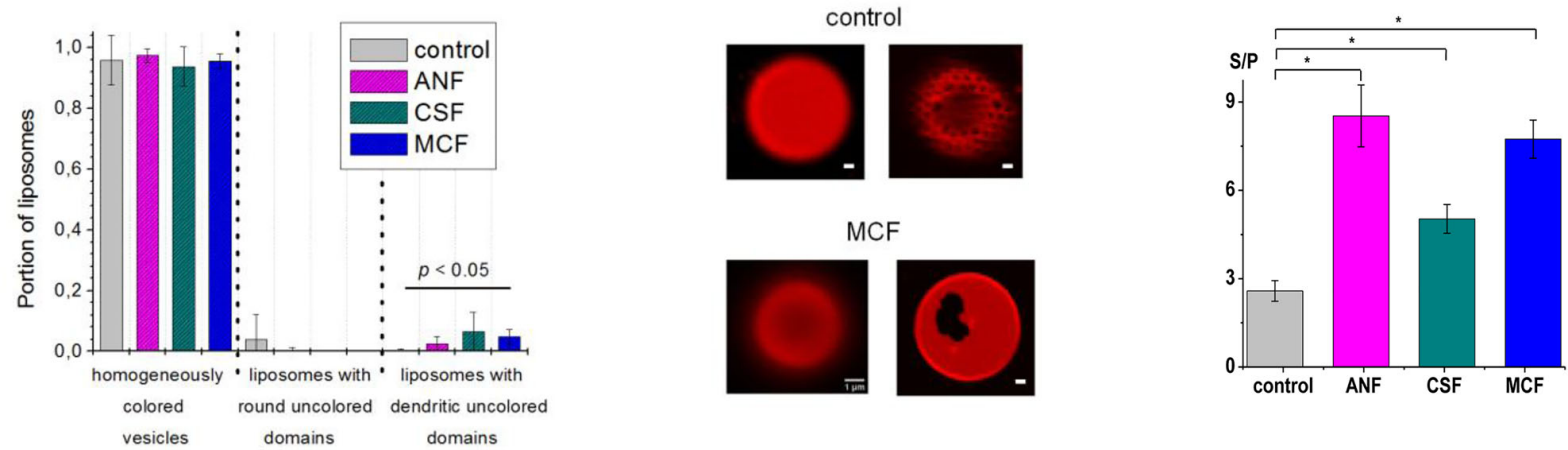
Effects of echinocandins on membrane lipid phase segregation. (Left panel) Bar chart showing the distribution of liposomes with different phase-separation scenarios (homogeneously colored vesicles and liposomes with round or dendritic uncolored domains) under control conditions (gray columns) and after addition of 50 µM ANF (magenta columns), 10 µM CSF (dark cyan columns), and 50 µM MCF (blue columns). The differences in uncolored lipid domain morphologies among conditions were statistically significant (*p* < 0.05, ANOVA). (*Middle panel*) Selected fluorescence microscopy images of GUVs formed from a DOPC:Chol (80:20 mol.%) mixture in the absence (control) and presence of MCF. The scale bar is 1 µm. Each sample contained 1 mol.% LR-PE to visualize ordered lipid domains. Regions labeled with LR-PE correspond to the liquid-disordered phase. Round and dendritic dark domains were attributed to higher and lower surface tensions at the boundaries between ordered and disordered phases, respectively. Experiments were conducted at room temperature. (Right panel) Mean ratio of the total area of uncolored domains to their overall perimeter (S/P). The difference between the control value and the S/P ratio in the presence of the tested echinocandins was statistically significant (*p* < 0.05, ANOVA with Tukey correction).

Thus, we hypothesized that the tested echinocandins cause differential phase disordering and should influence phase separation in the membrane. Therefore, we assessed changes in lipid domain organization in GUVs in the presence of ANF, CSF, and MCF by confocal fluorescence microscopy using LR-PE. This probe preferentially partitions into the fluid lipid phase and visualizes ordered lipid phases as uncolored domains. The vast majority of liposomes composed of a mixture of DOPC and Chol (80:20 mol.%) were homogeneously colored with LR-PE, indicating a liquid-disordered phase in both control experiments with pure liposomes and after echinocandin addition (Fig. 4, left panel). Figure 4 (middle panel) shows typical micrographs of homogeneously colored vesicles in the absence of echinocandins and in the presence of MCF. A few liposomes with multiple small, round, uncolored domains associated with the Chol-enriched ordered phase [36, 37] were detected in the control samples. Echinocandins had a distinctive effect on the phase-separation scenario. In addition to homogeneously colored vesicles, several percent of liposomes with large, uncolored, dendritic domains of uneven shape were observed in the presence of echinocandins. This was accompanied by a decrease in the total perimeter of the domains, which resulted from their fusion and subsequent enlargement. Figure 4 (right panel) presents the ratio between the total area of uncolored (ordered) domains in a liposome and their total perimeter. Echinocandins caused a significant increase in this ratio, clearly indicating an increase in line tension. We speculate that the lateral restructuring caused by echinocandins could have important consequences for the physiological functions of GS.

### Echinocandins increase membrane permeability

**Fig. 5:**
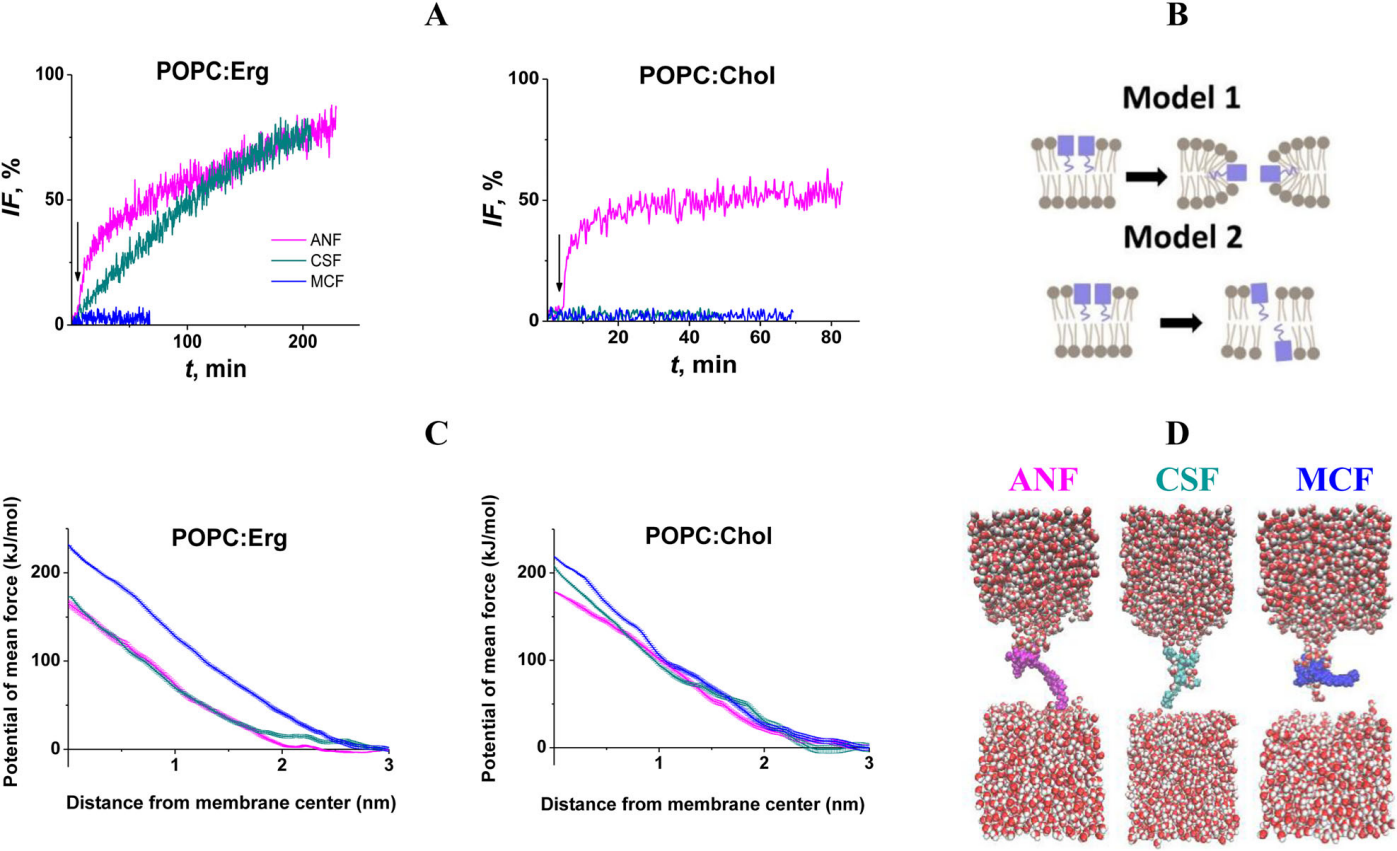
Echinocandin effects on membrane permeability. **A** Time dependence of relative fluorescence of calcein (IF, %) leaked from vesicles composed of POPC:Erg (67:33 mol.%) and POPC:Chol (67:33 mol.%) after addition of echinocandins. The moments of addition of ANF (*magenta curves*), CSF (*dark cyan curves*), and MCF (*blue curves*) into liposomal suspensions up to 50 μM are indicated by arrows. **B** Two models of peptide-induced calcein release, with the corresponding kinetic equations derived by Andersson et al. [39]. **C** Plots of the potential of mean force for ANF (*magenta curves*), CSF (*dark cyan curves*), and MCF (*blue curves*) molecules in lipid bilayers composed of POPC:Erg (67:33 mol.%) and POPC:Chol (67:33 mol.%). The free energy value was set to zero in the solvent region (3 nm from the membrane center). Error bars represent the standard error calculated by the bootstrap method. **D** Interactions between echinocandins and water during pulling MD simulations across the POPC:Erg (67:33 mol.%) bilayer (membrane removed for better visualization). Water molecules (atoms colored by element) were bound to the head groups of ANF (magenta), CSF (cyan), or MCF (blue) and were pulled together with the echinocandins.

The echinocandin-induced decrease in lipid order and packing could increase membrane permeability [38]. Indeed, ANF led to calcein efflux from liposomes prepared from POPC mixed with Erg (67:33 mol.%) or Chol (67:33 mol.%). CSF was active only in POPC:Erg (67:33 mol.%) vesicles; however, MCF, despite its pronounced effect on lipid melting thermograms, did not demonstrate significant activity in fluorescent marker release in any of the lipid models (Fig. 5A). These data indicate the selectivity of ANF and CSF for Erg-containing liposomes compared with Chol-enriched vesicles. This observation is consistent with the MD data, which showed that all echinocandins formed more interactions with Erg than with Chol (Table S3, Supplementary Information).

Andersson et al. [39] proposed two kinetic models for peptide-induced calcein release. The first model assumes pore formation by echinocandins, whereas the second suggests coupling of calcein leakage to peptide translocation between bilayer lipid leaflets. Figure 5B shows a schematic representation of these two models of echinocandin-induced calcein release. Applying Eqs. 3 and 4 (“Materials and methods”, Supplementary Information) to our data, we found that ANF-induced release could be interpreted in terms of Model 2 (the adjusted *R*^2^ parameter was 0.50 ± 0.06 for POPC:Chol and 0.76 ± 0.02 for POPC:Erg) (Table S4, Supplementary Information). Both models could be applied to approximate CSF-induced leakage from POPC:Erg liposomes (the adjusted *R*^2^ parameter was 0.91 ± 0.01); however, the fitting parameters for Model 1 were unrealistically large, and this model was therefore discarded. The fitting parameter *J*_2_, which is related to the rate of ANF-induced calcein transmembrane flow through Erg-containing membranes, was 2.1 times higher than that in Chol-containing membranes, indicating that ANF produced a greater perturbation in Erg-enriched membranes. The *v*_relax_ parameter was 18 times higher for ANF in Chol-containing membranes than in Erg-enriched membranes, indicating that the nonleaking equilibrium state was reached more rapidly in the former case. The fitting parameter *J*_2_ characterizing CSF-induced perturbation of Erg-containing membranes was four-fold higher, and *v*_relax_ was four-fold lower than those parameters for ANF, indicating greater membrane stress produced by CSF. The applicability of Eq. 4 to the experimental data indicates the possibility of echinocandin translocation across the membrane.

To assess this, we estimated the energy required for an echinocandin to cross the membrane by performing steered MD simulations and umbrella sampling (Fig. 5C). The energy differences for echinocandin localization between the water surface and the membrane center in Chol-containing membranes were 175.7 kJ/mol, 209.2 kJ/mol, and 217.6 kJ/mol for ANF, CSF, and MCF, respectively. For Erg-enriched bilayers, the differences were 163.2 kJ/mol, 171.6 kJ/mol, and 230.1 kJ/mol for ANF, CSF, and MCF, respectively. The difference between sterols for ANF and MCF was minimal (≈12 kJ/mol), whereas that for CSF was unexpectedly large (≈38 kJ/mol). These data clearly demonstrate that the penetration of echinocandins through membranes is associated with a large energy barrier due to the high energetic cost of transporting the molecule from the polar microenvironment to the center of the lipid bilayer. Notably, the umbrella sampling data (Fig. 5C) were consistent with the results of the release measurements (Fig. 5A). In particular, MCF, which did not cause calcein leakage, was characterized by the highest energy barrier for entry into the bilayer center. The energies of ANF translocation were minimal for both Erg- and Chol-containing membranes, and the drug produced calcein release from liposomes of both compositions. By contrast, the pronounced sterol dependence of the free energy of CSF transmembrane translocation was consistent with the inability of CSF to induce calcein efflux from POPC:Chol vesicles, together with significant drug-induced release from POPC:Erg liposomes. One way to reduce the energy barrier for drug translocation is to incorporate echinocandins into liposomal formulations.

The successful approximation of the experimental release curves by the translocation model equation does not exclude pore formation because the radius of an echinocandin-formed pore may be smaller than the hydrodynamic radius of calcein (0.74 nm) [40]. Moreover, when echinocandins were pulled across the membrane, they “dragged” several water molecules along with them (Fig. 5D). To test this possibility using a more precise technique, we assessed the effect of echinocandins on the ionic permeability of planar lipid bilayers of appropriate compositions and discovered for the first time that ANF, CSF, and MCF were able to form ion-permeable pores in POPC:Erg (67:33 mol.%) membranes (Fig. S4, insets, Supplementary Information). The cation–anion selectivity of the ANF, CSF, and MCF channels in POPC:Erg (67:33 mol.%) bilayers was also determined. The zero-current potential (*V*_rev_) was measured after the bilayer was modified by adding ANF, CSF, or MCF to the *cis* side only (Fig. S4, Supplementary Information). Figure S4 also demonstrates the differential cation–anion selectivity of pores induced by the different echinocandins. The *V*_rev_ values in the presence of ANF, CSF, and MCF were 25 ± 5, −12 ± 7, and 9 ± 5 mV, respectively. These reversal potentials corresponded to cation (Na^+^) transfer numbers of 0.7 ± 0.1, 0.4 ± 0.1, and 0.6 ± 0.1 for ANF, CSF, and MCF, respectively. The most probable explanation for the preferential cation selectivity of the ANF and MCF pores is related to the partial negative charges on the carbonyl groups that can orient toward the center of the ANF and MCF rings (Fig. 1). The weak anion selectivity of CSF pores may be explained by the presence of protonated amino groups in the side chains of the peptide core of CSF (Fig. 1).

Membrane bending rigidity controls pore formation by pore-forming proteins because membranes with lower bending moduli bend more easily, stabilizing ion-permeable pores [41]. Our analysis of lipid splay and tilt fluctuations showed that echinocandins lowered the membrane bending modulus across all tested systems (Fig. S5, Supplementary Information). Moreover, the introduction of 7.5 μM Lyso-PC increased the transmembrane currents induced by ANF, CSF, and MCF by 7-, 6.5-, and 11-fold, respectively (Fig. S6, Supplementary Information). The potentiation of echinocandin pore-forming ability by conical-shaped Lyso-PC is consistent with the toroidal pore model and supports the hypothesis of membrane bending during echinocandin pore formation.

### Echinocandin-loaded lipid formulations are characterized by increased activity against resistant pathogenic strains

Encapsulation of antifungals into liposomal drug formulations may improve their pharmacological properties [42]. For instance, more efficient penetration of the liposomal form of fluconazole inhibited the growth of biofilm-forming *C. albicans* isolates [43]. Liposomal formulations of amphotericin B have been developed to address the low aqueous solubility and high toxicity of this drug. Additionally, rapid accumulation at infection sites (including biofilms) has been observed for these formulations [44]. Liposome-based approaches are not only intended to facilitate delivery, decrease toxicity, and improve bioavailability; lipid formulations of chemotherapeutic agents have also been shown to combat anticancer drug resistance [45]. To date, nanosomal prototypes of drug formulations have been developed and evaluated only for ANF [46]. Although the minimum inhibitory concentration (MIC) against *C. albicans* was equivalent to that of the free drug (probably due to low ANF loading), the nanosomal formulation exhibited superior biofilm disruption, reducing the fungal burden by 99%. Thus, the development of liposomal formulations of echinocandins could help circumvent fungal resistance associated with biofilm formation.

To enhance the membrane activity of echinocandins and increase their selectivity toward fungal membranes, the drugs were loaded into POPC:Chol (67:33 mol.%) liposomes at lipid:drug molar ratios of 4:1. The concentrations of ANF, CSF, and MCF in the liposomal formulations were determined using molar absorption coefficients in DMSO. The encapsulation efficiencies of ANF, CSF, and MCF into POPC:Chol liposomes were approximately 70%, 45%, and 80%, respectively. To independently confirm that the lipid-associated drug formulations were present as lipid vesicles, electron microscopy was performed. Figure S7A (Supplementary Information) presents typical electron microscopy images of echinocandin-loaded liposomes. The diameter of unmodified POPC:Chol liposomes without echinocandins was 125 ± 30 nm (data not shown). Liposomal formulations containing ANF, CSF, and MCF showed uniform size distributions, with mean diameters of 130 ± 40, 146 ± 30, and 140 ± 35 nm, respectively (Fig. S7B, Supplementary Information). The similarity of these values within the experimental error suggests that ANF, CSF, and MCF do not significantly alter liposome size.

**Fig. 6:**
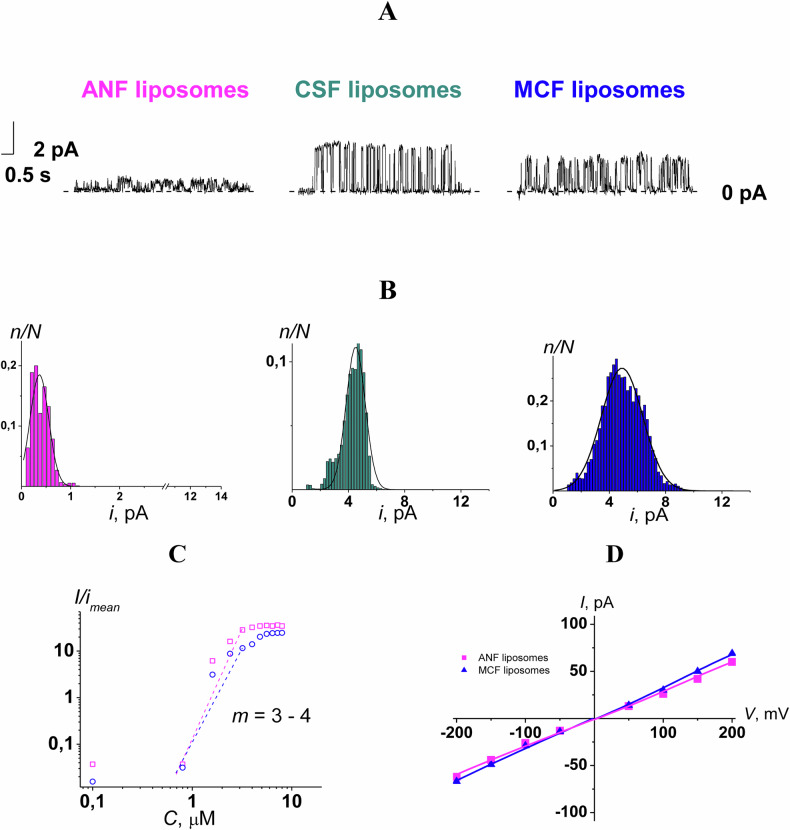
Pore-forming activity and increased potency of lipid formulations of echinocandins. **A** Typical recordings of current fluctuations corresponding to openings and closures of single ion-permeable pores induced by the addition of liposomes composed of POPC:Chol (67:33 mol.%) loaded with different echinocandins. Planar lipid bilayers were formed from POPC:Erg (67:33 mol.%) and bathed in 0.15 M NaCl (10 mM HEPES, pH 7.4). **B** Corresponding current transition histograms at *V* = 100 mV. **C** Dependence of the ratio between the steady-state current (*I*) flowing through bilayers treated with liposomes loaded with ANF (magenta symbols) or MCF (blue symbols) and the mean amplitude of single echinocandin-induced pores (*i*_mean_) on the concentration (*C*) of lipopeptides in bilogarithmic coordinates at *V* = 100 mV. **D** Dependence of the steady-state current flowing through bilayers treated with liposomes loaded with ANF or MCF (*I*) on transmembrane voltage (*V*). The concentrations of ANF (magenta curve) and MCF (blue curve) in the *cis*-side bathing solution were estimated to be 7 μM.

We next assessed the ability of echinocandin liposomal formulations to increase the permeability of planar lipid bilayers mimicking fungal cell membranes. Figure 6A shows a typical recording of current fluctuations corresponding to openings and closings of single ion-permeable pores induced by the addition of ANF-, CSF-, and MCF-loaded liposomes to an aqueous solution bathing POPC:Erg (67:33 mol.%) bilayers. The figure also presents corresponding histograms showing the distribution of current amplitudes of single pores induced by the various echinocandins. Pore amplitude increased in the order ANF (mean current, *i*_mean_ ≈ 0.4 pA) < CSF ≈ MCF (*i*_mean_ ≈ 4.5–4.9 pA) (Fig. 6B). The radius of the equivalent conducting cylinders was estimated to be approximately 0.1 and 0.3 nm, respectively, which is smaller than the hydrodynamic radius of calcein.

One-sided addition of ANF- and MCF-loaded liposomes to the POPC:Erg membrane bathing solution increased the macroscopic membrane conductance in a dose-dependent manner. By contrast, subsequent addition of CSF-loaded liposomes to the membrane bathing solution did not lead to a significant increase in the transmembrane current, and only a few single pores were observed regardless of the lipopeptide concentration (Fig. S8, Supplementary Information). Figure 6C shows bilogarithmic plots of the dependence of the steady-state transmembrane current flowing through POPC:Erg membranes treated with ANF- and MCF-loaded liposomes at 100 mV on drug concentration. The slopes of the linear regression models describing the growth region of these curves for ANF and MCF were close to 3–4 and did not significantly depend on echinocandin type. This suggests that at least three to four echinocandin molecules participate in forming the conductive subunit. Figure 6D shows the *I(V)* characteristics of POPC:Erg membranes treated with ANF- and MCF liposomes. The *I(V)* curves were symmetrical and nearly linear.

**Fig. 7:**
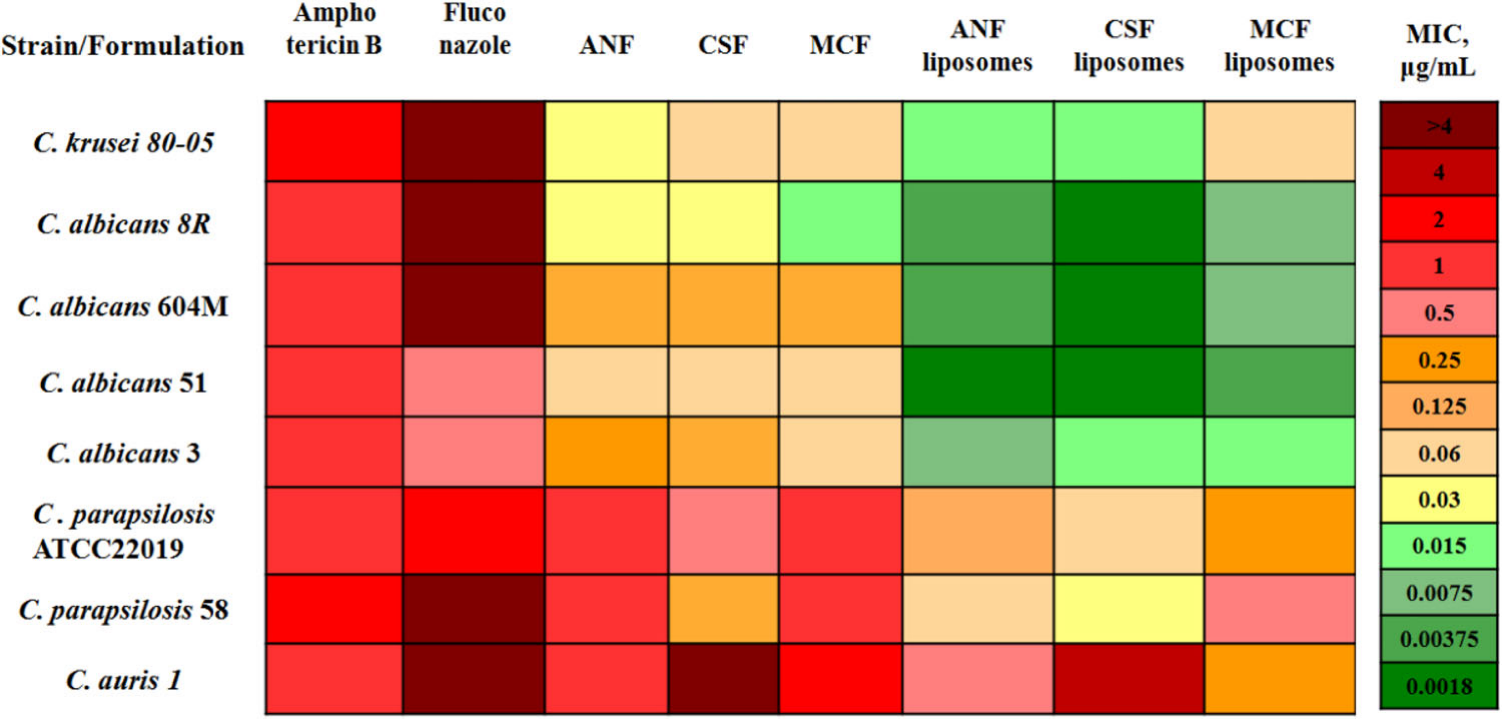
Antifungal tests. Heatmap showing the antimicrobial activity of the tested echinocandins and their liposomal formulations against a range of fungal strains.

Next, we studied the activity of echinocandin-loaded liposomes against a broad panel of fungal pathogens. Clinical isolates of *Candida* species resistant to fluconazole (*C. krusei* 80-05, *C. albicans* 8 R, *C. albicans* 604 M, *C. albicans* 51, *C. albicans* 3) and to echinocandins (*C. parapsilosis* ATCC 22019, *C. parapsilosis* 58, *C. auris* 1) were used. The strain *C. parapsilosis* ATCC 22019, in particular, is known for its reduced echinocandin susceptibility due to a point mutation in the FKS1p gene encoding GS [47]. Liposomes without echinocandins did not demonstrate antifungal activity against the tested microorganisms at the concentrations used. Figure 7 presents a heat map of the antimicrobial activity of echinocandin-loaded liposomes against *Candida* spp. Amphotericin B, fluconazole, and echinocandins (ANF, CSF, and MCF) dissolved in DMSO/medium were used as controls. As a rule, liposomal preparations of echinocandins were characterized by lower MICs than the free echinocandins against all tested fungal strains, including those tolerant or resistant to conventional echinocandins. Analysis of Fig. 7 showed that the ratio between the MICs of echinocandins and their liposomal forms did not significantly correlate with the MICs of the free echinocandins (Fig. S9A, Supplementary Information). This indicates that potentiation of echinocandin antifungal activity by inclusion of the drugs in liposomal form was not directly determined by the susceptibility of the strain to the conventional drug formulation. Therefore, we conclude that incorporation of echinocandins into liposomal formulations may enhance their antimicrobial activity through increased membrane activity. Moreover, we found that the relative increase in echinocandin antifungal activity produced by lipid formulation depended notably on the *Candida* species. Figure S9B (Supplementary Information) shows that the average ratio between the MICs of conventional and liposomal echinocandins decreased in the following order: *C. albicans* > *C. parapsilosis* > *C. krusei* ≈ *C. auris*. Previous studies have reported significant differences in the lipid compositions of different *Candida* species. In particular, the relative level of Erg decreases in a similar order [48, 49] (Fig. S9B, Supplementary Information). This tendency may be explained by the effect of echinocandins on Erg-enriched domains in which GS is localized, or by the dependence of drug pore-forming ability on Erg content. Interestingly, the mean ratios between the MICs of echinocandins and their liposomal forms were higher for strains characterized by a higher relative content of anionic phospholipids and saturated fatty acid tails (Fig. S9B, Supplementary Information). This observation may indicate a possible dependence of echinocandin membrane activity on these factors, which requires further study. Figure S10 (Supplementary Information) presents the effect of incorporating negatively charged POPS into the POPC membrane on the efficiency of echinocandins in inducing calcein leakage. POPS increased CSF activity but decreased ANF activity. MCF did not induce marker release from vesicles composed of either pure POPC or POPS-containing liposomes. The sensitivity of both tested *C. parapsilosis* strains to CSF was higher than to ANF and MCF (Fig. 7), whereas the opposite tendency was observed for *C. auris* 1. Figure S9B (Supplementary Information) shows that the ratio between anionic and neutral lipid content is higher in *C. parapsilosis* than in *C. auris*. Thus, the difference in sensitivity may be explained by potentiation and inhibition of the membrane activity of CSF and ANF, respectively, with increasing anionic lipid content.

## Discussion

The findings presented here elucidate the complex interplay between echinocandins and lipid membranes and reveal mechanisms underlying their antifungal efficacy and selectivity. By integrating biophysical, computational, and cell assay approaches, this study advances our understanding of how echinocandins disrupt membrane organization and how lipid-based formulations can enhance their therapeutic potential.

MCC microdomains serve as protective islands that stabilize associated proteins and maintain fungal virulence [50]. Although GS is not confined to MCC and exhibits dynamic mobility, point mutations that alter the protein composition of these microdomains trigger membrane reorganization, leading to abnormal cell wall synthesis, impaired morphogenesis, and increased *C. albicans* susceptibility to stress [51, 52]. To investigate the underlying mechanisms, we examined the domain-remodeling effects of echinocandins on simplified model membranes. The observed broadening of DSmC transition peaks (∆*T*_*b*_) (Fig. 2A) and reduced acyl-chain order (S_CD_) in Erg-enriched systems (Fig. S3, Supporting Information) reflect echinocandin-induced membrane fluidization, which destabilizes lipid microdomains. Erg favors echinocandin insertion, as evidenced by MD simulations showing spontaneous partitioning of ANF and MCF into Erg-rich bilayers (Table S2, Supplementary Information). These findings support the hypothesis that echinocandins target GS by perturbing its lipid microenvironment in addition to directly inhibiting the enzyme.

A study using fluorescent echinocandins revealed that CSF accumulates in *Candida* vacuoles rather than in the plasma membrane, as observed for ANF [53]. Our DSmC (Fig. 2A) and MD data (Table S2, Supplementary Information) help explain this observation by demonstrating the lower ability of CSF to embed in the membrane from aqueous solution. Encapsulation promotes bilayer integration of CSF and might therefore be used as a strategy to overcome this limitation.

Amphipathic antimicrobial peptides with antifungal activity primarily exert their effects through pore-forming mechanisms that disrupt fungal membrane integrity. For example, histatin-5 forms ion-permeable pores in *C. albicans* mitochondria, leading to cell death [54]. Similarly, recombinant actifensin and defensin-d2 exert antifungal activity against *C. albicans* and *P. aeruginosa* through pore formation [55]. Based on the structural similarities between echinocandins and known pore-forming peptides, we assumed that these drugs might also possess pore-forming activity. Calcein release assays revealed that ANF and CSF induced membrane perturbation (Fig. 5A), consistent with their low free-energy barriers for membrane penetration (Fig. 5C). By contrast, despite its significant membrane-disordering effect in DSmC and MD studies, MCF was unable to induce leakage (Fig. 5A), which correlates with its high translocation energy. Unexpectedly, all three echinocandins formed stable ion-permeable pores in Erg-rich lipid bilayers (Fig. 6A and Fig. S4, Supporting Information). This previously unreported activity may have been overlooked because its contribution to fungicidal effects is minimal compared with the primary action of GS inhibition.

The interplay between echinocandin-induced lipid disordering, alterations in phase segregation, and pore formation can be understood as an integrated process underpinning their antifungal action. Our DSmC and MD data showed that echinocandins induce lipid disordering, and these effects are strongly influenced by the lipid phase state. Lipid disordering modulates key physical properties such as bending rigidity, spontaneous curvature, and elastic moduli, which in turn influence line tension and elastic deformation at domain boundaries, affecting the size, stability, and tendency for phase segregation [56]. Confocal microscopy experiments clearly showed that echinocandin-induced increases in line tension favor the formation of fewer but larger domains, whereas lower line tension in control membranes stabilizes many smaller domains. This effect may be explained by the observation that echinocandins reduce the difference between the spontaneous curvatures of ordered and disordered lipid phases, as discussed above. MD simulations and experimental data demonstrate that multicomponent lipid membranes exhibit a broad range of bending rigidities depending on lipid composition and phase state, with Chol-enriched ordered phases showing up to a 3- to 5-fold higher bending modulus than disordered phases [57]. Echinocandins induce lipid disordering, which fluidizes the membrane and decreases the bending modulus (Fig. S5, Supplementary Information). This reduction in rigidity is critical because more flexible membranes bend more easily, thereby lowering the energetic barrier for pore nucleation and stabilizing ion-permeable pores—a mechanism supported by recent studies [41]. Thus, the reduced bending modulus observed in the presence of echinocandins may promote pore stabilization, increasing membrane permeability and enhancing antifungal activity.

Liposomal formulations of echinocandins reduce the energy barrier for drug translocation and increase pore-forming capacity, resulting in improved membrane integration. Several studies have shown that lipid formulations improve biofilm penetration and reduce MICs for resistant *Candida* strains [42, 43, 45]. Notably, the species-dependent efficacy of liposomal echinocandins (Fig. 7 and Fig. S9B, Supplementary Information), based on fungal membrane composition—particularly Erg content—supports a membrane-mediated mechanism of action for these formulations.

This study provides a mechanistic rationale for optimizing echinocandin formulations. Liposomal delivery enhances membrane disruption, potentially mitigating fungal resistance caused by GS mutations. However, the reliance on simplified model membranes (e.g., DPPC:sterol systems) warrants caution. Future studies should examine echinocandin interactions with asymmetric, multicomponent fungal membranes containing sphingolipids and other phospholipids that influence drug partitioning and pore stability. Additionally, in vivo studies are required to assess the pharmacokinetic advantages of echinocandin-loaded liposomes and their ability to target fungal infections in complex biological environments.

## Conclusion

Our study provides insights into the critical role of membrane activity in the action of well-known antifungal echinocandins. Echinocandins were shown to significantly affect phase separation in model lipid membranes. Considering that the main echinocandin target, GS, is embedded in Erg-enriched lipid rafts [18], reorganization of lipid domains by echinocandins may perturb GS function.

For the first time in more than 20 years of clinical echinocandin use, dramatic changes in membrane permeability upon adsorption of echinocandins and the ability of these antibiotics to form ion-permeable pores in model lipid membranes have been demonstrated. These findings reveal additional modes of antifungal action of echinocandins that are not directly related to GS inhibition. We also showed that the membrane activity of echinocandins can be greatly enhanced by their incorporation into liposomes composed of phosphatidylcholine and Chol. The potentiation of membrane activity upon incorporation of echinocandins into lipid vesicles, the preferential selectivity of liposomal preparations for Erg-containing fungal membranes, and the induction of pore formation led to a significant decrease in MIC values against various clinical isolates, including strains with reduced susceptibility to clinically important echinocandins. Thus, the present study not only demonstrates alternative mechanisms of action of echinocandins and the possibility of directly targeting pathogen membranes to overcome resistance, but also provides a foundation for future clinical trials of lipid formulations of echinocandins.

## Materials and methods

### Materials

All chemicals used were of reagent grade. Synthetic POPC, POPS, DPPC, DOPC, LR-PE, Lyso-PC, and Chol were obtained from Avanti Polar Lipids, Inc. (Pelham, AL). CSF (≥97% [HPLC]), ANF (≥97% [HPLC]), MCF (≥97% [HPLC]), amphotericin B, fluconazole, Erg, calcein, DMSO, Sephadex G-50, Triton X-100, sorbitol, EDTA, NaCl, HEPES, KOH, and NaOH were purchased from Sigma-Aldrich Company Ltd. (Gillingham, United Kingdom). Stock solutions of echinocandins were prepared in DMSO. Distilled water was used in all experiments.

### Choice of lipid systems

The choice of lipid types was deliberate and based on the specific requirements and constraints of each experimental method. For DSmC, only lipids with high melting temperatures were used, such as DPPC (melting temperature 41.5 °C) and its mixtures with a low percentage of sterols. Increasing sterol content above 20% causes peak broadening, which complicates data interpretation. By contrast, calcein release assays require lipids with melting temperatures well below room temperature to ensure that the bilayer remains in a fluid phase; therefore, POPC and its mixtures with sterols or POPS were employed. Similarly, confocal microscopy was performed using DOPC:Chol mixtures because systems containing POPC instead of DOPC do not exhibit phase separation relevant for domain imaging [58]. Regarding sterol composition, vesicles used in the confocal microscopy experiments were composed of DOPC:Chol because GUVs prepared with DOPC:Erg mixtures generally exhibit smaller sizes, which limits their suitability for evaluating phase segregation due to reduced resolution and associated analytical complications. Electrophysiological measurements were conducted at room temperature under lipid requirements similar to those of the release assay. The use of lipids with high melting temperatures is infeasible for these measurements because the resulting bilayers would not be in the fluid phase. For MD simulations, any membrane system may be selected to probe molecular mechanisms and allow comparison across methods. Accordingly, different membrane compositions were used depending on the property of interest and their relevance to the experimental systems.

### Preparation of echinocandin-loaded liposomes

The technique described by Yamskov et al. [59], with modifications for the formation of liposomes loaded with echinocandins, was used. Lipid mixtures (67 mol.% POPC and 33 mol.% Chol), with or without ANF, CSF, or MCF at lipid:drug molar ratios of 4:1, were suspended in a mixture of chloroform and methanol (67:33 vol.%). The resulting solution was evaporated in a vacuum rotary evaporator at room temperature for 120 min. The lipid film was then dispersed in buffer (0.15 M NaCl, 10 mM HEPES, 1 mM EDTA, pH 7.4) and exposed to ultrasound for 10 min. The concentrations of ANF, MCF, and CSF in the liposomal preparations were determined following an approach by [60] and using the molar absorption coefficients obtained in DMSO or methanol at the wavelength of maximum absorbance. For comparison, UV absorbance spectra of the echinocandins in buffer solution were also measured. Absorbance spectroscopy was performed on a Fluorat-02-Panorama spectrofluorometer (Lumex, Saint Petersburg, Russia). All samples were scanned from 200 to 400 nm in 1-nm steps. The concentrations of ANF, CSF, and MCF in the liposome preparations were 34 ± 7, 21 ± 4, and 40 ± 6 µM, respectively. Formulations without echinocandins were also scanned to subtract for the turbidity of the liposomal suspension. Encapsulation efficiency of echinocandins in liposomes was calculated as the ratio between the encapsulated and the added drug. To characterize the size of the echinocandin liposomal preparations, electron microscopy was performed. Negative staining was carried out using a 1% aqueous solution of uranyl acetate for 20 s. Lipid preparations were placed on copper grids coated with a collodion film substrate. Electron micrographs were obtained using a Libra 120 transmission electron microscope (Carl Zeiss, Jena, Germany). The sizes of the lipid structures were averaged from two independent experiments for each tested system and are presented as mean ± standard deviation (*p* ≤ 0.05).

### DSmC

GUVs were prepared using pure DPPC or mixtures of DPPC:Chol (85:15 mol.%) and DPPC:Erg (85:15 mol.%), with or without echinocandins (at lipid:drug molar ratios of 10:1), according to the electroformation method using Vesicle Prep Pro® (Nanion Technologies, Munich, Germany) (standard protocol: 3 V, 10 Hz, 58 min, 55 °C). The liposome suspensions contained 5 mM lipids and were buffered with 5 mM HEPES–KOH at pH 7.4. The echinocandins ANF, CSF, and MCF from stock solutions in DMSO were added to ready-to-use lipid suspension aliquots at lipid:echinocandin molar ratios of 50:1, 25:1, and 10:1, or added during liposome preparation at a ratio of 10:1. The suspensions were then heated and cooled at constant rates of 0.2 and 0.3 °C/min, respectively, using a µDSC 7EVO microcalorimeter (Setaram, Caluire-et-Cuire, France). The reversibility of thermal transitions was assessed by reheating the sample immediately after the cooling step from the previous scan.

Lipid thermograms were characterized by the pre-transition temperature attributed to the mobility of the choline polar head group (only in the case of DPPC), melting temperature (*T*_m_, defined as the temperature at which excess heat capacity reaches a maximum), and the enthalpy of the main phase transition (Δ*H*_cal_, the area under the main peak). The sharpness of the gel-to–liquid-crystalline phase transition was expressed as Δ*T*_b_, defined as the temperature difference between the upper (onset) and lower (completion) boundaries of the main phase transition.

Deconvolution analysis of the main peak in the presence of echinocandins was performed using Calisto software. Separation of overlapping peaks was based on the application of Gaussian or Fraser–Suzuki (asymmetric) functions. The melting temperature of each *i*-peak component, *T*_*m_i*_, and the percentage contribution of *i*-peak component to the total area, ${p}_{i}=\frac{\varDelta {H}_{i}}{\varDelta {H}_{\mathrm{cal}}}\cdot 100 \%$, were determined. The calculated signal was fitted to the experimental data using nonlinear optimization (Marquardt algorithm).

At least two independent experiments were performed for each lipid composition–echinocandin combination to ensure reproducibility of the results.

### MD simulation

Model membranes were assembled using the CHARMM-GUI Membrane Builder [61] and contained the following numbers of lipid and drug molecules (Table 1):

**Table 1:** Composition of bilayers used for MD simulations.

Experimental design	Membrane composition	Number and placement of echinocandin molecules
APL/S_CD_ measurements	1) 120 DPPC 2) 136 DPPC, 24 Erg 3) 136 DPPC, 24 Chol 4) 120 POPC 5) 108 POPC, 52 Erg 6) 108 POPC, 52 Chol	12 and 16 echinocandin molecules inserted into membranes containing 120 and 160 lipids, respectively
Incorporation experiments	1) 100 POPC, 50 Erg 2) 100 POPC, 50 Chol	4 molecules in solution
Bonds assessment	1) 100 POPC, 50 Erg 2) 100 POPC, 50 Chol	4 molecules inserted into the membrane
Umbrella sampling	1) 40 POPC, 20 Erg 2) 40 POPC, 20 Chol	1 molecule in solution

The aqueous solution was ionized with 0.15 M NaCl, except in experiments with DPPC:Chol and DPPC:Erg systems, which were ion-free to mimic the DSmC conditions. Topological parameters for echinocandins were generated using CGenFF [62].

MD simulations were performed using GROMACS 2023.2 [63] with the CHARMM36m all-atom force field [64]. Energy minimization was carried out using the steepest descent algorithm. A six-step equilibration procedure was applied, gradually releasing position restraints on lipid molecules (total equilibration time ≈ 2 ns). All simulations were conducted at constant temperatures of 25 °C (298 K) or 50 °C (323 K) and a pressure of 1 bar using the Nosé–Hoover thermostat and semi-isotropic pressure coupling with a C-rescaled barostat [65, 66]. The temperature and pressure coupling time constants were 1 and 5 ps, respectively. Long-range electrostatic interactions were treated using the Particle Mesh Ewald method with a short-range cutoff of 1.2 nm [67]. Van der Waals interactions were calculated using the shifted Lennard–Jones potential with a general cutoff of 1.2 nm and a shifting cutoff of 1.0 nm. Trajectories for production simulations were recorded every 10 ps, whereas trajectories for steered simulations and umbrella sampling were recorded every 1 ps. Production simulations were performed for 100 ns. Incorporation experiments were performed in four replicas.

The APL in POPC membranes was 63.1 Å^2^, in agreement with experimental (62.7 Å^2^ at 20 °C, 64.3 Å^2^ at 30 °C [68]) and MD data (63.5 Å^2^ at 303.15 K [69]).

Steered MD simulations were performed to enhance echinocandin penetration through membranes. This approach is widely used for characterizing molecule–membrane interactions and obtaining free-energy profiles [70–72]. In this study, a harmonic potential with a force constant of 500 kJ/mol/nm^2^ was applied between the center of mass of the echinocandin molecule and the membrane along the *z*-axis. The frame in which the echinocandin molecule was positioned 3 nm above the membrane center was selected from the production run and used as the starting point for a 50-ns pulling simulation, allowing the molecule to penetrate the lipid bilayer smoothly. Successive umbrella-sampling windows were run for 50 ns at distances ranging from 0.0 to 3.0 nm from the membrane center along the *z*-axis at intervals of 0.2 nm, resulting in 16 simulation windows. The weighted histogram analysis method (WHAM) was then applied to the resulting data to obtain the potential of mean force [73]. Standard errors were estimated using the bootstrap method [74]. Visualization was performed using VMD [75]. Interactions between molecules were assessed using the Hydrogen Bonds plugin in VMD and were defined as interactions with donor–acceptor distances less than 6 Å (including both hydrogen bonds and van der Waals contacts). APL and S_CD_ were calculated using MEMBPLUGIN [76] and are presented as averages from 20 ns until the end of the simulations. To analyze the structural organization of the lipid bilayer, mass density profiles were calculated using the gmx density tool. The lipid molecules were partitioned into distinct groups for phospholipid headgroups and hydrocarbon tails using a custom index file to evaluate their specific distributions along the bilayer normal. The bending modulus was calculated using a Python script described in [57]. Internal lipid vectors were first defined, and their tilt angles relative to the membrane normal were measured. Splay angles were then determined between these tilt vectors for neighboring lipid pairs within a defined radius. Statistical analysis of these angles allowed calculation of the potential of mean force describing the energy cost of splay, which was then fitted to a quadratic function to determine the bending modulus.

### Confocal fluorescence microscopy

GUVs were formed from DOPC:Chol (80:20 mol.%) by the electroformation method, as described above (standard protocol: 3 V, 10 Hz, 58 min, 45 °C). The lipid stock solution was prepared in chloroform. Labeling was performed by adding the fluorescent lipid probe LR-PE (1 mol.%). The resulting aqueous liposome suspension, containing 0.8 mM lipid and 1.5 M sorbitol, was divided into 30-μL aliquots. Control liposome samples did not contain echinocandins. ANF and MCF were introduced into liposome suspensions from DMSO stock solutions at concentrations up to 50 μM; CSF was tested at 10 μM. Liposome suspensions containing echinocandins were equilibrated for 30 min at room temperature (25 °C ± 2 °C). Samples were observed using a standard microscopy preparation, with 10 μL of the liposome suspension placed on a microscope slide and covered with a coverslip. GUV membranes were imaged with an oil immersion objective (65 × /1.4 HCX PL) using an Olympus FV3000 microscope (Hamburg, Germany). The temperature during observation was controlled by air heating or cooling using a thermally insulated chamber. LR-PE was excited at a wavelength of 543 nm (helium–neon laser). LR-PE preferentially partitions into the liquid-disordered phase and is excluded from ordered lipid phases [77]. Surface tension forces, which tend to shorten the boundary between ordered and disordered phases, produce round liquid-ordered regions that appear as uncolored by LR-PE round domains. The DOPC:Chol (80:20 mol.%) GUV membranes were predominantly visually homogeneous and remained in the liquid-disordered phase. A small number of DOPC:Chol vesicles possessed higher sterol content and contained round uncolored domains that could be attributed to the liquid-ordered lipid phase, as described previously [36, 37, 78]. The number of GUVs within a single field of view, with or without visible phase separation of different types (small uncolored round domains and large uncolored domains with complex shapes), was counted. Several neighboring fields of view were analyzed.

The proportion of vesicles with different phase-separation scenarios in each tested system was calculated as the ratio of GUVs with or without particular domains to the total number of GUVs. Experiments were performed in eight replicates with a mean of 125 liposomes per condition. Differences among groups were assessed using one-way ANOVA with a significance level of *p* ≤ 0.05.

The quantitative impact of echinocandins on line tension was evaluated by determining the ratio of the total area of uncolored domains to their overall perimeter (*S/P*) for each individual liposome and averaging the values across the entire vesicle population.

### Calcein leakage assay

The fluorescence of calcein released from unilamellar lipid vesicles was used to monitor membrane permeabilization induced by echinocandins. Liposomes were prepared from POPC, POPC:Erg (67:33 mol.%), POPC:Chol (67:33 mol.%), and POPC:POPS (50:50 mol.%) by extrusion using an Avanti Polar Lipids mini-extruder (Pelham, AL). The lipid stock in chloroform was dried under a gentle stream of nitrogen. The dry lipid film was then hydrated with calcein-containing buffer (35 mM calcein, 10 mM HEPES–NaOH, pH 7.4). The suspension was subjected to five freeze–thaw cycles and passed 13 times through a 100-nm nucleopore polycarbonate membrane. Calcein not entrapped in vesicles was removed by gel filtration using a Sephadex G-50 column, replacing the external buffer with a calcein-free solution (0.15 M NaCl, 1 mM EDTA, 10 mM HEPES–NaOH, pH 7.4). The initial lipid concentration in suspension was 3 mM. Calcein encapsulated in vesicles fluoresced poorly because of strong self-quenching at millimolar concentrations, whereas the fluorescence of released calcein in the surrounding solution correlated with membrane permeabilization induced by echinocandins.

Echinocandins were added to calcein-loaded liposomes from stock solutions (4 mM in DMSO). Time-dependent calcein fluorescence dequenching induced by 50 μM echinocandins was measured over 50 min, with at least three replicates for each lipid–echinocandin combination.

The degree of calcein release was determined at room temperature using a Fluorat-02-Panorama spectrofluorometer (Lumex, Saint-Petersburg, Russia). A 10-mm quartz cuvette was used to measure calcein leakage from liposomes after the addition of echinocandins. The excitation wavelength was 490 nm, and the emission wavelength was 520 nm. Triton X-100 was added to each sample to a final concentration of 1 vol.% to achieve complete disruption of unilamellar lipid vesicles, and fluorescence intensity was measured after release of the total amount of calcein from the liposomes.

The relative intensity of calcein fluorescence (*IF*, %) was used to describe the dependence of liposome permeabilization on echinocandin type and membrane composition. *IF* was calculated using the following formula:

1 $$IF=\frac{I-I0}{\frac{Imax}{0.9}-I0}\cdot 100 \% ,$$

where *I* and *I*_0_ are the calcein fluorescence intensities in the sample in the presence and absence of echinocandin, respectively, and *I*_max_ is the maximal fluorescence measured after liposome lysis with Triton X-100. A factor of 0.9 was introduced to account for dilution of the sample by detergent.

The kinetics of calcein release were analyzed using the models proposed by Andersson et al. [39]. To assess the first model (Model 1), which assumes peptide-induced pore formation, the following equation was applied:

2 $$\frac{IF(t)}{I{F}_{max}}=1-\exp \left(\frac{{J}_{1}}{{v}_{fluct}^{2}}\left(1-{v}_{fluct}{t-e}^{-{v}_{fluct}t}\right)\right)$$

where *IF*_max_ is the maximum leakage in the presence of echinocandin, *J*_1_ is a fitting parameter that depends on both pore kinetics and transmembrane calcein flow, and *v*_fluct_ is the rate at which the pore fluctuates between open and closed states.

The second model (Model 2), describing peptide-induced membrane stress or perturbation, predicts the following dependence of leakage on time:

3 $$\frac{IF(t)}{I{F}_{max}}=1-\exp \left({J}_{2}\left({e}^{-{v}_{relax}t}-1\right)\right)$$

where *IF*_max_ is the maximum leakage in the presence of echinocandin, and *J*_2_ and *v*_relax_ are related to the rate of calcein flow and the rate of membrane reorganization leading to a nonleaking equilibrium state, respectively.

Experimental data were fitted to Eqs. (2 and 3) using Origin 8.1 software. The fitting quality was assessed using the adjusted *R*^2^ parameter.

### Registration of ion-permeable pores in planar lipid bilayers

Virtually solvent-free bilayers were prepared according to the monolayer-opposition technique [79] on a 50-µm-diameter aperture in a 10-µm-thick Teflon film separating the Teflon chamber into two compartments (*cis* and *trans*). The aperture was pretreated with hexadecane. Planar lipid membranes were composed of POPC:Erg (67:33 mol.%) and bathed in either symmetric salt solutions (0.15 M NaCl, 10 mM HEPES, pH 7.4) or asymmetric salt solutions (0.025 M NaCl, 10 mM HEPES, pH 7.4 in the *cis* compartment and 0.15 M NaCl, 10 mM HEPES, pH 7.4 in the *trans* compartment). After the membrane had fully formed and stabilized, pure echinocandins dissolved in DMSO or echinocandin-loaded lipid formulations were added to the *cis* compartment to final concentrations of 0.1–10 μM active drug. Echinocandin-free liposomal formulations or DMSO alone did not affect membrane stability or permeability. Measurements of transmembrane current (*I*) and applied transmembrane voltage (*V*) were performed using Ag–AgCl electrodes with 2 M KCl–1.5% agarose bridges. At positive voltages, the *cis* compartment was positive relative to the *trans* compartment. All experiments were conducted at room temperature.

An Axopatch 200B amplifier (Molecular Devices, Sunnyvale, CA, USA) was used in voltage-clamp mode to measure current. Signals were digitized using a Digidata 1440 A interface (Molecular Devices, Sunnyvale, CA, USA) at a sampling frequency of 5 kHz with 1 kHz low-pass filtering. Data analysis was performed using pClamp 10 (Molecular Devices) with filtering using an eight-pole Bessel 100-kHz filter and Origin 8.0 software (OriginLab Corporation, Northampton, MA, USA).

The total number of events used to calculate the mean current amplitude of single ion-permeable pores induced by echinocandins ranged from 1000 to 4000.

The dependence of the steady-state echinocandin-induced transmembrane current (*I*) on the lipopeptide concentration (*C*) in bilogarithmic coordinates at *V* = 100 mV was used to evaluate slope factors corresponding to the number of molecules (*m*) forming a single conductive unit.

Two-sided addition of Lyso-PC (from stock aqueous millimolar solutions) to a final concentration of 7.5 μM in the bathing solution was used to modulate the pore-forming activity of echinocandins.

The transport numbers of cations (*t*^+^) and anions (*t*^*–*^ = *1* – *t*^+^) were determined as previously described [80]:

4 $${V}^{\mathrm{rev}}=\frac{{kT}}{e}\left(1-{2{\rm{t}}}^{+}\right)\mathrm{ln}\left(\frac{{C}_{1}}{{C}_{2}}\right)$$

where ${V}^{\mathrm{rev}}$ is the reversal potential (the voltage at which the transmembrane current equals zero), *k* is the Boltzmann constant, *T* is the thermodynamic temperature, *e* is the elementary charge, and ${C}_{1}$ and ${C}_{2}$ are the concentrations of the NaCl solutions in the *cis* and *trans* compartments, respectively.

### Antifungal tests

#### Test microorganisms

Clinical isolates of fluconazole-resistant strains *C. albicans* 604M, *C. albicans* 8R, *C. albicans* 51, *C. albicans* 3, *C. parapsilosis* 58L, and *C. auris* 1 used for screening antifungal compounds were kindly provided by A.B. Kulko (Moscow Government Health Department Scientific and Clinical Antituberculosis Center, Moscow). *C. parapsilosis* ATCC 22019 was purchased from the American Type Culture Collection (ATCC, Manassas, VA, USA) and used as a quality control strain for antifungal susceptibility testing.

#### Sample preparation

Samples of echinocandin-loaded liposomes were diluted in RPMI 1640 medium according to the desired concentration of the active component. Amphotericin B, fluconazole, ANF, CSF, and MCF, used as controls, were dissolved in DMSO to a concentration of 10,000 μg/mL and then diluted in the nutrient medium. The concentration range for the loaded liposomes was 2.0–0.0009 μg/mL (calculated based on the echinocandin concentration), and the concentration range for the control preparations of pure echinocandins was 8.0–0.00375 μg/mL. Liposomes not loaded with echinocandins were analyzed using undiluted samples, and antimicrobial activity was assessed using the highest dilution factor at which no visible growth occurred. These preparations did not exhibit notable antifungal activity against the tested *Candida* strains.

#### Assay configuration

Antifungal activity was assessed using the serial microdilution method in nutrient broth according to EUCAST definitive document E.DEF 7.3.2 (April 2020), Method for the determination of broth dilution MICs of antifungal agents for yeasts. The nutrient medium RPMI 1640 (with L-glutamine and pH indicator but without bicarbonate) was prepared from dry powder according to the manufacturer’s instructions (Sigma, Lot SLBZ6264, St. Louis, MO, USA) with the addition of glucose to a final concentration of 2% and 3-(*N*-morpholino)propanesulfonic acid (MOPS; PanEco, Russia) to a final concentration of 0.165 mol/L. The pH was adjusted to 7.0 with 1 N sodium hydroxide.

For each *Candida* culture, single colonies grown on Sabouraud agar for 48 h were suspended in phosphate-buffered solution, and the inoculum density was determined according to the McFarland standard (5 × 10^6^ CFU/mL) using a Den-1B densitometer (BioSan, Riga, Latvia). Following serial dilutions of the tested samples in 96-well plates (Medpolymer, Russia), the inoculum was added to obtain a final density of approximately 2 × 10^5^ CFU/mL.

The plates were incubated without shaking at 35 °C ± 2 °C under aerobic conditions. Antifungal activity was visually assessed after 24 and 48 h. The MIC value corresponded to the lowest concentration at which no visible growth of the test microorganism was observed.

### Data and statistical analysis

Results are presented as either mean ± SD (mean ± SE in the case of *S*/*P*-value) or mean only, wherever applicable. Differences between groups were assessed using a one-way ANOVA test with a significance level of *p* ≤ 0.05. *p*-values are labeled according to the convention of ns > 0.05, *p ≤ 0.05, **p ≤ 0.01, and ***p ≤ 0.001. The deconvolution analysis of the differential scanning microcalorimetry thermograms was performed using the Calisto software. Statistical analysis and fitting were performed using Origin 8.1 software. Statistical details are provided in the figure legends and in the “Materials and methods” where applicable.

## Supplementary information

Supplementary Information

## Data Availability

All analyzed and generated data during the current study are included in the article and Supplementary Information. Further information is available from the corresponding author on reasonable request.
