# Supplementary material for: Echinocandins have an alternative mode of action on biomimetic membranes that is not directly related to the functioning of (1,3) beta-glucan synthase
Source: Cell Death Discov. 2026 Apr 28;12:274. doi: 10.1038/s41420-026-03133-8 (PMC13265816; doi:10.1038/s41420-026-03133-8)
Supplement: Supplementary file 1 — Supplementary Information [file 41420_2026_3133_MOESM1_ESM.pdf]

Supplementary Information

Echinocandins have an alternative mode of action on biomimetic membranes that is not directly related to the functioning of (1,3) beta-glucan synthase

Anna I. Malykhina[1,§], *Svetlana S. Efimova*[1,§], *Natalia E. Grammatikova*[2], *Anna N. Tevyashova*[3], *Andrey E. Shchekotikhin*[2], *and Olga S. Ostroumova*[1]

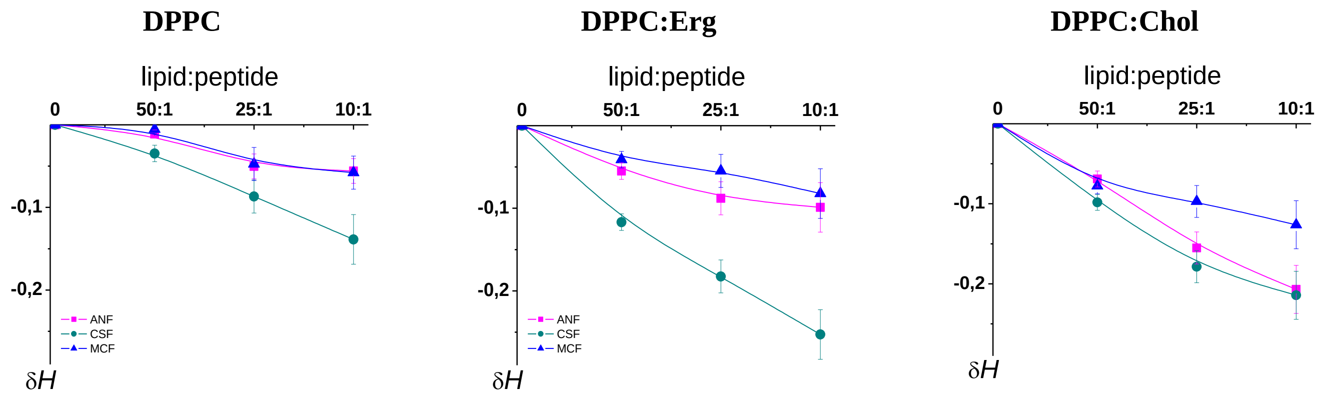

Figure S1. The dependences of the relative changes in enthalpy of the main phase transition (δ*H*) of DPPC (*left panel*), DPPC:Erg (85:15 mol.%) (*medium panel*), and DPPC:Chol (85:15 mol.%) (*right panel*) on molar ratio of lipid to ANF (*magenta curves*), CSF (*dark cyan curves*), and MCF (*blue curves*).

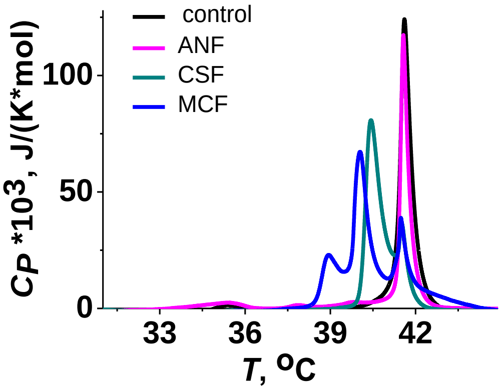

Figure S2. Heating thermograms of unilamellar liposomes composed of pure DPPC (control, *black curves*) and mixtures of DPPC with ANF (*magenta curves*), CSF (*dark cyan curves*), and MCF (*blue curves*) at a lipid:echinocandin ratio of 10:1. Echinocandins were added at the moment of liposome formation.

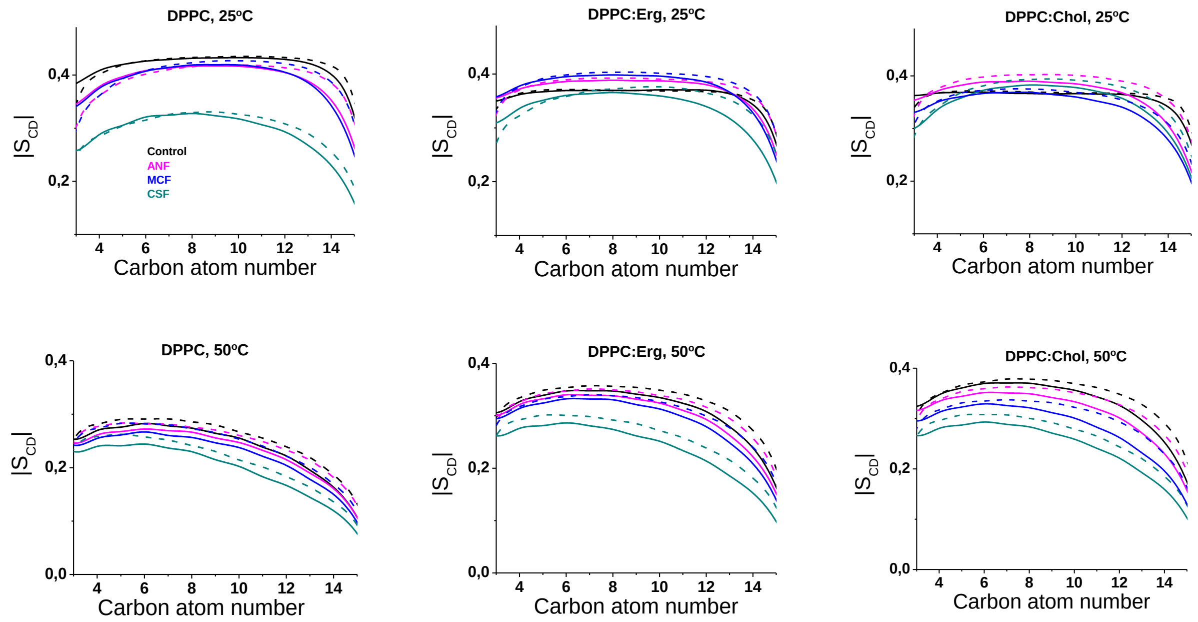

Figure S3. Order parameter S_{CD} for the acyl chains of DPPC in membranes composed of DPPC (*left panel*), DPPC:Erg (85:15 mol.%) (*medium panel*), and DPPC:Chol (85:15 mol.%) (*right panel*) in the absence (control, *black curves*) and presence of ANF (*magenta curves*), CSF (*dark cyan curves*), and MCF (*blue curves*), at lipid:echinocandin ratios of 10:1, based on MD data at 25°C (*upper panel*) and 50°C (*lower panel*). The *sn*-1 and *sn*-2 acyl chains of DPPC are marked as solid and dashed lines, respectively.

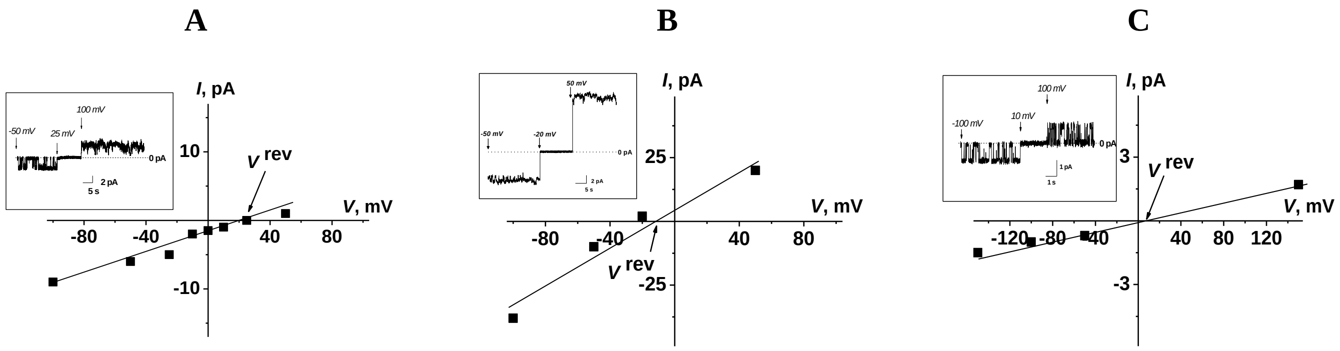

Figure S4. Cation-anion selectivity of transmembrane pores induced by (**A**) ANF, (**B**) CSF, and (**C**) MCF. *I*–*V* curves of membranes modified with sufficient amounts of echinocandins (*cis*-side-added only) are presented. Arrows indicate the reversal potential (V_{rev}). *Inset*: Typical records presenting current fluctuations corresponding to openings and closures of (**A**) ANF-, (**B**) CSF-, and (**C**) MCF-induced pores at different transmembrane voltages (voltage values are shown above the arrows). The membranes were composed of POPC:Erg (67:33 mol.%) and bathed in 0.025 M NaCl (10 mM HEPES, pH 7.4) at the *cis* side and 0.15 M NaCl (10 mM HEPES, pH 7.4) at the *trans* side.

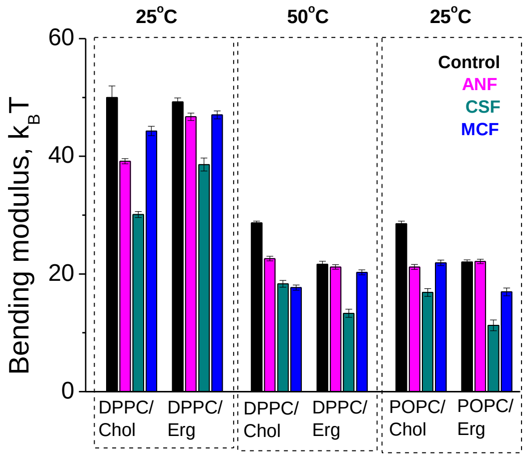

Figure S5. Bending modulus of different membranes (DPPC:Chol (85:15 mol.%), DPPC:Erg (85:15 mol.%), POPC:Chol (67:33 mol.%), POPC:Erg (67:33 mol.%)) in the absence (control – *black*) and presence of echinocandins (ANF – *magenta*, CSF – *dark cyan*, MCF – *blue*) calculated based on MD simulation at temperatures 25°C and 50°C.

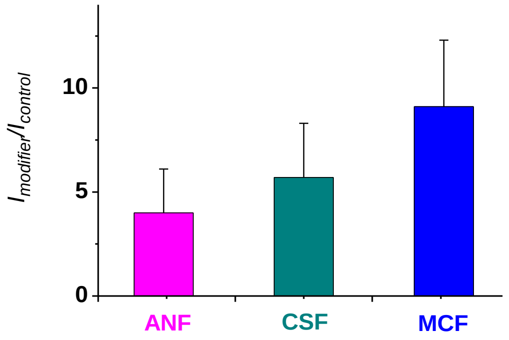

Figure S6. The effects of 7.5 μM Lyso-PC on the pore-forming activity of ANF, CSF, and MCF. The membranes were composed of POPC:Erg (67:33 mol.%) and bathed in 0.025 M NaCl (10 mM HEPES, pH 7.4) at the *cis* side and 0.15 M NaCl (10 mM HEPES, pH 7.4) at the *trans* side. The transmembrane voltage was 100 mV.

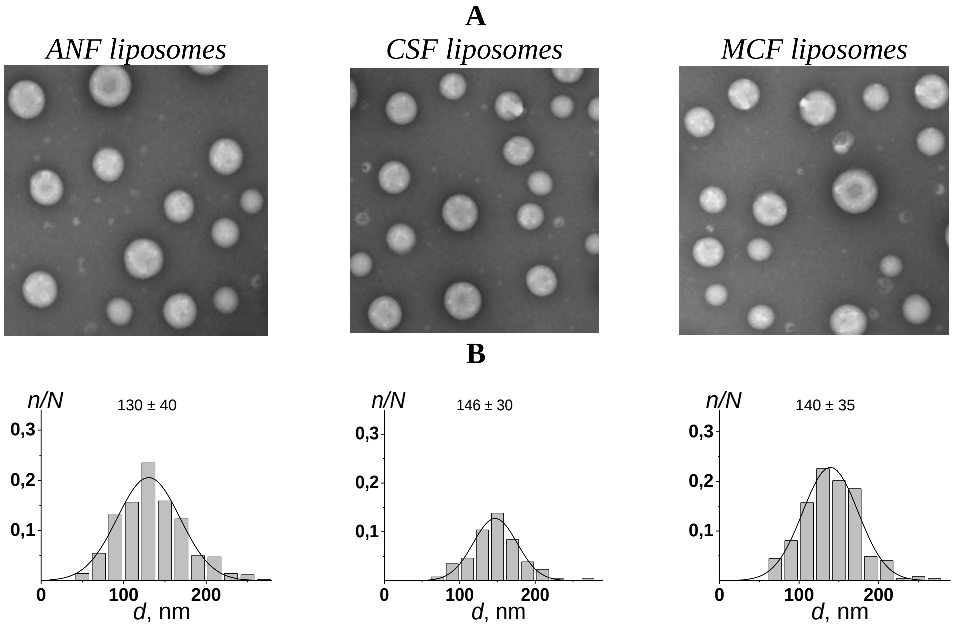

Figure S7. Characterization of echinocandin-loaded lipid formulations. (**A**) Typical electron microscopy images of liposomes loaded with different echinocandins. The size of each image is equal to 800 × 800 nm. (**B**) Histograms of diameter (*d*) of echinocandin-loaded liposomes. The mean values obtained by fitting histograms by the normal distribution are presented at the top of each figure. The differences in diameter of liposomes in various formulations were not statistically significant ($p < 0.05$, ANOVA).

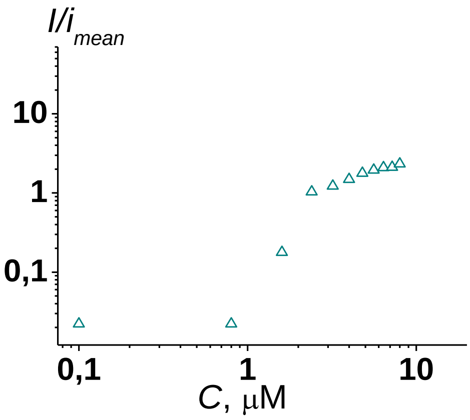

Figure S8. The dependence of the ratio between steady-state current (*I*) flowing through bilayers treated with liposomes loaded with CSF and mean amplitude of single CSF-induced pores (i_{mean}) on the lipopeptide concentration (*C*) in bilogarithmic coordinates at $V = 100$ mV.

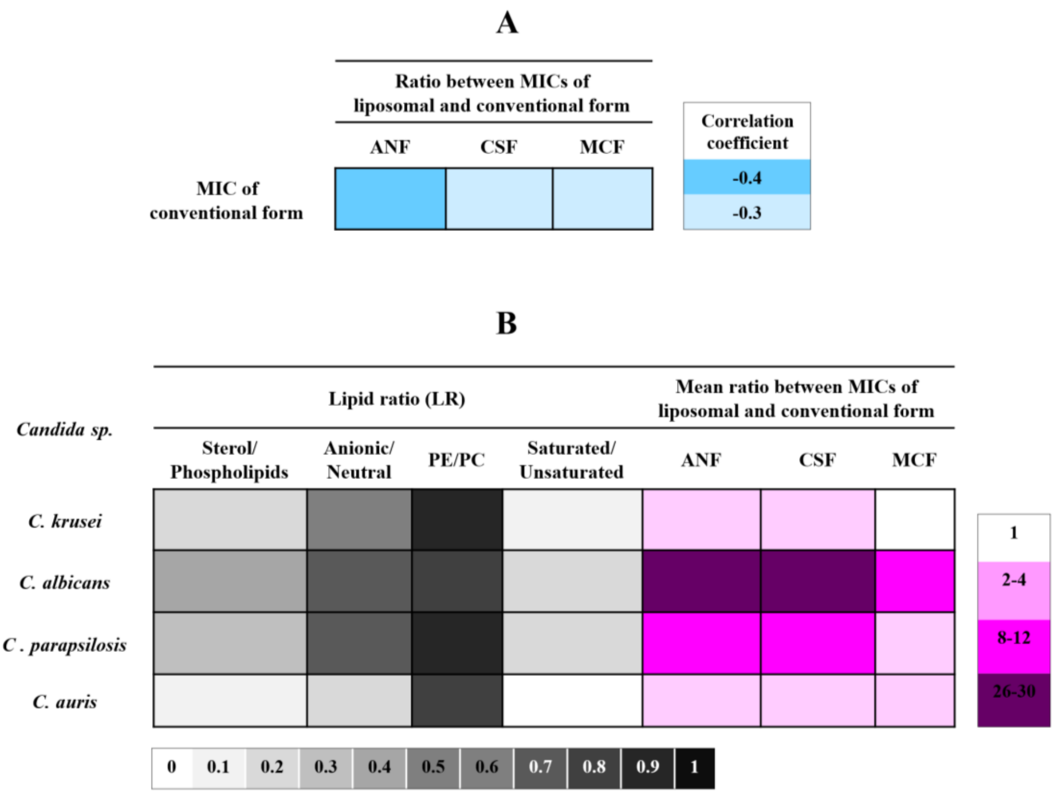

Figure S9. (**A**) Correlation between the efficiency of conventional echinocandins against tested *Candida* clinical isolates (MIC) and relative increase in the echinocandin potency produced by drug inclusion into liposomal form (ratio between MICs of echinocandin in liposomal and conventional forms). Correlation analysis was performed using data presented in Fig. 7. (**B**) Possible relation between the changes in lipid composition of various *Candida* species and a relative increase in echinocandin mean potency against these species produced by drug inclusion into liposomal form. Ratio between contents of Erg and phospholipids (Sterol/Phospholipids), anionic and neutral phospholipids (Anionic/Neutral), phosphatidylethanolamine and phosphatidylcholine species (PE/PC), and saturated and unsaturated fatty acids (Saturated/Unsaturated) for *C. krusei*, *C. albicans*, and *C. parapsilosis* were estimated based on lipidomic data by [48]. Lipid ratios for azole-resistant *C. auris* were presented according to [49].

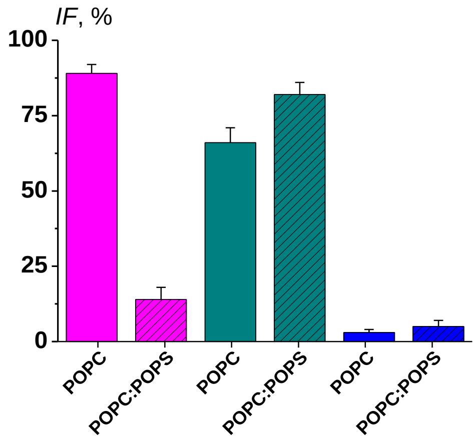

Figure S10. Bar chart showing maximal relative intensity of calcein fluorescence (*IF*, %) released from POPC and POPC:POPS (50:50 mol.%) liposomes after addition of 50 μM ANF (*magenta columns*), CSF (*dark cyan columns*), and MCF (*blue columns*).

Table S1. Echinocandin effects on APL (Å^2) in membranes of different compositions assessed by MD. A lipid:echinocandin molar ratio was equal to 10:1.

echinocandin	**DPPC**		**DPPC:Erg (85:15 mol.%)**		**DPPC:Chol (85:15 mol.%)**	
	25°C	*50°C*	*25°C*	*50°C*	*25°C*	*50°C*
control	50.5 ± 0.9	61.2 ± 2.1	45.5 ± 0.6	51.5 ± 0.9	43.9 ± 0.7	50.3 ± 1.6
ANF	58.5 ± 1.0	65.6 ± 1.5	49.8 ± 0.9	55.0 ± 0.8	49.5 ± 0.9	53.9 ± 1.0
CSF	66.2 ± 1.3	70.6 ± 1.5	55.1 ± 1.0	61.8 ± 0.9	57.5 ± 0.7	62.0 ± 0.9
MCF	57.5 ± 1.4	65.3 ± 1.8	49.9 ± 1.3	56.1 ± 0.9	51.3 ± 1.7	56.8 ± 1.1
	**POPC**		**POPC:Erg (67:33 mol.%)**		**POPC:Chol (67:33 mol.%)**	
control	63.6 ± 1.3		35.2 ± 0.6		34.0 ± 0.5	
ANF	67.1 ± 1.6		37.1 ± 0.7		37.3 ± 0.5	
CSF	73.7 ± 1.3		41.5 ± 0.8		40.5 ± 0.5	
MCF	67.6 ± 1.3		38.0 ± 0.7		37.2 ± 0.6	

Table S2. Number of echinocandin molecules that were spontaneously inserted into the lipid bilayer of appropriate composition from aqueous solution during 100 ns of MD simulation. Four echinocandin molecules were added in the solution at the initial moment. The experiment was repeated 4 times. The table contains the mean number ± SD.

**Membrane composition**	**ANF**		**CSF**		**MCF**	
	Erg	***Chol***	***Erg***	***Chol***	***Erg***	***Chol***
**POPC:sterol (67:33 mol.%)**	1.0 ± 0.8	0.5 ± 0.6	0.25 ± 0.5	0 ± 0	0.75 ± 0.5	0 ± 0

Table S3. Mean numbers of bonds (hydrogen bonds and Van-der-Waals interactions) up to 6 Å between echinocandins and other molecules in 100 ns of simulation. Four molecules of each echinocandin were initially embedded into POPC:sterol (67:33 mol.%) membranes. For water, only hydrogen bonds were counted.

**Bonds to**	**ANF**		**CSF**		**MCF**	
	**POPC:Erg**	**POPC:Chol**	**POPC:Erg**	**POPC:Chol**	**POPC:Erg**	**POPC:Chol**
sterol	52.4 ± 13.5	27.7 ± 12.7	97.2 ± 14.4	84.3 ± 14.2	43.6 ± 10.7	36.7 ± 9.7
POPC	239.7 ± 21.4	261.2 ± 21.9	214.5 ± 21.1	221.2 ± 22.9	206.8 ± 21.8	243.9 ± 23.8
water	25.4 ± 5.0	26.9 ± 4.7	24.8 ± 4.4	24.9 ± 4.5	29.2 ± 4.8	27.8 ± 4.6
echinocandin	20.6 ± 7.1	20.4 ± 7.9	39.7 ± 11.1	49.1 ± 11.2	44.6 ± 12.5	36.2 ± 10.2

Table S4. Parameters characterizing the dependence of echinocandin-induced calcein leakage on time, fitting by Eq. 4.

**parameter**	**POPC:Erg (67:33 mol.%)**			**POPC:Chol (67:33 mol.%)**		
	ANF	*CSF*	*MCF*	*ANF*	*CSF*	*MCF*
IF_{max}, %	73 ± 4	76 ± 1	1 ± 1	47 ± 4	1 ± 1	1 ± 1
J_2	1.43 ± 0.33	5.95 ± 0.07	N/A	0.68 ± 0.12	N/A	N/A
V_{relax}, min^{-1}	0.008 ± 0.006	0.002 ± 0.001	N/A	0.18 ± 0.06	N/A	N/A
Adjusted R^2	0.76 ± 0.02	0.91 ± 0.01	N/A	0.50 ± 0.06	N/A	N/A
